# Biomarkers of nanomaterials hazard from multi-layer data

**DOI:** 10.1038/s41467-022-31609-5

**Published:** 2022-07-01

**Authors:** Vittorio Fortino, Pia Anneli Sofia Kinaret, Michele Fratello, Angela Serra, Laura Aliisa Saarimäki, Audrey Gallud, Govind Gupta, Gerard Vales, Manuel Correia, Omid Rasool, Jimmy Ytterberg, Marco Monopoli, Tiina Skoog, Peter Ritchie, Sergio Moya, Socorro Vázquez-Campos, Richard Handy, Roland Grafström, Lang Tran, Roman Zubarev, Riitta Lahesmaa, Kenneth Dawson, Katrin Loeschner, Erik Husfeldt Larsen, Fritz Krombach, Hannu Norppa, Juha Kere, Kai Savolainen, Harri Alenius, Bengt Fadeel, Dario Greco

**Affiliations:** 1 Institute of Biomedicine, University of Eastern Finland, Kuopio, Finland; 2 Faculty of Medicine and Health Technology, Tampere University, Tampere, Finland; 3 BioMediTech Institute, Tampere University, Tampere, Finland; 4 Institute of Biotechnology, University of Helsinki, Helsinki, Finland; 5 Finnish Hub for Development and Validation of Integrated Approaches (FHAIVE), Tampere, Finland; 6 Institute of Environmental Medicine, Karolinska Institutet, Stockholm, Sweden; 7 Finnish Institute of Occupational Health, Helsinki, Finland; 8 National Food Institute, Technical University of Denmark, Kgs. Lynby, Denmark; 9 Turku Bioscience Centre, University of Turku, and Åbo Akademi University, Turku, Finland; 10 Department of Medical Biochemistry and Biophysics, Karolinska Institutet, Stockholm, Sweden; 11 Department of Pharmaceutical and Medicinal Chemistry, Royal College of Surgeons in Ireland, Dublin, Ireland; 12 Department of Biosciences and Nutrition, Karolinska Institutet, Huddinge, Sweden; 13 Institute of Occupational Medicine, Edinburgh, UK; 14 Soft Matter Nanotechnology Laboratory, CIC biomaGUNE, San Sebastian, Spain; 15 Leitat Technological Center, Terrassa, Spain; 16 School of Biological and Marine Sciences, University of Plymouth, Plymouth, UK; 17 Division of Toxicology, Misvik Biology, Turku, Finland; 18 Centre for BioNano Interactions, School of Chemistry and Chemical Biology, University College Dublin, Dublin, Ireland; 19 Walter Brendel Centre of Experimental Medicine, Ludwig-Maximilians-Universität München, Munich, Germany; 20 Department of Bacteriology and Immunology, University of Helsinki, Helsinki, Finland

**Keywords:** Bioinformatics, Predictive markers, Nanotoxicology

## Abstract

There is an urgent need to apply effective, data-driven approaches to reliably predict engineered nanomaterial (ENM) toxicity. Here we introduce a predictive computational framework based on the molecular and phenotypic effects of a large panel of ENMs across multiple in vitro and in vivo models. Our methodology allows for the grouping of ENMs based on multi-omics approaches combined with robust toxicity tests. Importantly, we identify mRNA-based toxicity markers and extensively replicate them in multiple independent datasets. We find that models based on combinations of omics-derived features and material intrinsic properties display significantly improved predictive accuracy as compared to physicochemical properties alone.

## Abstract (web summary)

Nanomaterials have a range of potential applications, however, toxicity remains a concern, limiting application and requiring extensive testing. Here, the authors report on a predictive framework made using a range of tests linking materials properties with toxicity, allowing the prediction of toxicity from physiochemical and biological properties.

## Introduction

Advances in molecular and cellular biology, along with technological improvements in assay development, are changing the paradigm of chemical safety assessment^1^. These developments are also being increasingly adopted for the safety assessment of engineered nanomaterials (ENMs)^2,3^. In particular, there is a shift from descriptive toxicology towards a mechanism-based predictive assessment of chemicals and ENMs. Computational approaches in which intrinsic properties of the toxicant are used as predictors of toxicity have been traditionally exploited in the context of quantitative structure–activity relationship (QSAR) modeling. However, these methods, although valid in principle, do not provide information concerning the biological mechanism of action of chemicals. An important change of paradigm in chemical safety assessment concerns the quantitative analysis of molecular (mechanistic) and functional (phenotypic) effects occurring at multiple levels of the biological organization. To this end, systems biology approaches are applied in order to discover biomarkers of exposure and uncover the mechanism of action of chemical substances^4^. Omics technologies, including transcriptomics and proteomics, are predicted to have a key role in future risk assessment strategies and these methodologies have been extensively used in recent years for the assessment of the biological responses of ENMs in vitro and in vivo^5,6^. The combination of different omics-based methods provides a powerful way to evaluate the biological impact of low-dose exposure to toxicants^7^, which are necessary to replicate real-life situations for humans and the environment^8^. Hence, Toxicogenomics provides new insight into the chemical–biological interactions, which goes beyond the traditional structure–activity relationships. Recently, significant efforts have been initiated in order to explain intermediate mechanistic aspects of chemical exposure through the adoption of adverse outcome pathways (AOP) and the definition of biomarkers of the mechanism of action of toxicants. Such efforts are coordinated in Europe in the form of multiple EU-funded projects and initiatives coordinated by the Joint Research Center (JRC) of the European Commission, in the USA by the activities of the Environmental Protection Agency (EPA), as well as globally under the Environmental, Health and Safety (EHS) program of the Organization for Economic Co-operation and Development (OECD) expert groups such as the Advisory Group on Molecular Screening and Toxicogenomics (EAGMST).

While biomarkers selected based on a priori knowledge have already been used as proxies of apical events in the context of large screening programs including recent U.S. inter-agency collaborations^9,10^, toxicogenomics can help to identify new mechanistic biomarkers in a hypothesis-free fashion^2,11^.

One of the main challenges in nanosafety concerns the lack of understanding of the rules that govern the biological effects of different ENMs^12^. Likewise, specific biomarkers of ENM exposure remain to be identified. Previous work demonstrated the utility of a multipronged assessment of ENMs, using a panel of cell types and cytotoxicity assays reflective of different endpoints, and has shown that it is possible to identify ENMs with similar patterns of biological activity across different cell types^13^. However, the latter study did not provide information on (predictive) biomarkers of ENM toxicity.

**Fig. 1:**
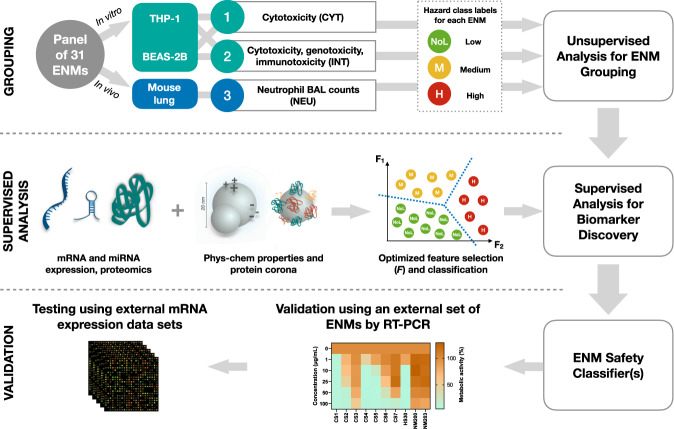
The experimental and computational approach taken to develop the ENM safety classifier. The set of 31 ENMs comprises common nanomaterials with different core chemistries, sizes, shapes, and surface modifications. THP-1 and BEAS-2B cells were exposed to a low-dose (EC10) of the ENMs alongside the in vivo exposures in mice. The panel of 31 ENMs were evaluated for their hazard based on cytotoxicity (CYT) and the combination of cytotoxicity, genotoxicity, and immunotoxicity (INT) in vitro as well as neutrophil (NEU) in vivo. Unsupervised learning techniques were then applied to group the ENMs based on the assessed toxicity endpoints. Next, feature selection and classification algorithms were used to identify subsets of molecular features (or biomarker models) and physicochemical properties that can distinguish ENMs with different hazard labels (NoL, Med, High). The identified biomarker models were validated by RT-PCR using an external panel of ENMs. Finally, RF-based classifiers that were trained on mRNA-based biomarker models were tested using publicly available mRNA expression profiles from various ENM exposures.

In this work we implement a multi-layered, omics-driven systems toxicology approach for ENM grouping and prioritization. The main objective is to derive a set of omics-based biomarkers that, in combination with the intrinsic properties of ENMs, could predict their hazard potential. To this end, data obtained using different in vitro and in vivo assays are homogenized and integrated in order to group the ENMs into three hazard classes (i.e., low or no hazard, intermediate hazard, and high hazard). Then, feature selection algorithms and Random Forest (RF) classifiers are used to identify sets of composite (or synergistic) biomarkers able to distinguish the three hazard classes (Fig. 1). The models selected from different data layers, including omics-based data as well as data on intrinsic properties of ENM, are evaluated alone and in combination to test whether the integration of heterogeneous data can improve the predictions.

## Results

### ENMs, apical toxicity, and mechanistic data overview

The present study focuses on a comprehensive toxicity assessment of 31 industrially relevant ENMs. The set of ENMs covers eight different chemistries, spanning both metal and metal oxide nanomaterials (including semiconductor crystals) as well as carbonaceous materials, i.e., multi-walled carbon nanotubes and nanodiamonds (NDs) with three different surface modifications, i.e., amino/ammonium groups, carboxyl/carboxylate groups and poly(ethylene glycol) (PEG)-terminated surfaces, and unmodified ENMs (designated as “core” ENMs), along with variations in size (e.g., 5 nm versus 20 nm Au particles) and shape (i.e., spherical versus rod-shaped TiO_2_ particles). We focused on industrially relevant ENMs and the role of surface modifications. Although it is impossible to cover all available ENMs in the context of a single study due to limited resources, those selected here are representative of a range of different physicochemical properties and hazard potentials for robust predictions. Detailed information on the synthesis can be found in ref. ^14^. Moreover, the Expanded Methods in the Suppl. Information file provides details on the 31 ENMs and the experimental protocols. A rigorous grid design was applied in order to allow direct evaluation of the effects of the chemistry and surface properties, as well as their combination. The multi-omics datasets included global expression levels of mRNA, miRNA, and proteins from two human cell lines (monocyte-like THP-1 cells and bronchial epithelial BEAS-2B cells), mRNA expression from mouse lung tissues, protein corona profiles, and comprehensive characterization of all the ENMs (Supplementary Table 1). THP-1 cells are commonly used as a model for evaluating the cyto- and immunotoxicity of ENMs^15,16^, while BEAS-2B cells are a preferred model for the in vitro assessment of pulmonary toxicity including the potential genotoxicity of ENMs^17,18^. The multi-omics-based analysis was based on an equipotent, low-concentration exposure (EC_10_). The EC_10_ was identified by means of concentration-response studies carried in the THP-1 and BEAS-2B cell lines, as the dose (for each of the 31 EMNs) that elicited 10% cell death. At this subtoxic concentration, the mechanism of action measurements do not mirror the toxicity, since toxic phenotypes are not yet exacerbated. In fact, while the molecular alterations (e.g., at the transcriptomics level) can be intuitively used as a relatively direct measure of toxicity when the exposure doses are high enough to observe toxic phenotypes, they become the indication of important intermediate underlying mechanisms when observed at low, subtoxic, concentrations. Moreover, high doses that allow direct observation of acute toxicity in vitro and/or in vivo are often unable to mimic real-life exposure scenarios. For these reasons, we decided to measure the molecular mechanism of action (transcriptomics, proteomics, etc.) in experimental conditions (EC_10_) in which they would not bluntly mirror toxicity phenotypes, but in which they would instead allow the identification of early mechanistic biomarkers of toxicity, as previously described^19–21^.

### Grouping of ENMs into hazard categories

The data collected from the in vitro assays (cytotoxicity, genotoxicity, and immunotoxicity) was homogenized by applying a point-based conversion system that resulted in a general toxicity score between 1 (no toxicity) and 6 (highest toxicity). Supplementary Tables 2–5 describe the criteria to apply the point-based conversion system, while Supplementary Tables 6–8 illustrate some examples of the homogenization results. The Bayesian information criterion (BIC) was used to determine the number of clusters for each toxicity grouping (Supplementary Fig. 1). The BIC was computed by varying the number of clusters between 2 and 6. The analysis showed that 2, 3, and 4 are reasonable numbers of groups since they represent a good compromise between the BIC values and the model stability. Based on the result of the BIC analysis, three hazard categories were defined: (1) no-to-low (NoL), (2) medium (M), and (3) high (H) hazard. For the sake of clarity and brevity, only the results using these three hazard categories are shown here. However, results using 2 and 4 hazard categories are also reported in Supplementary Fig. 7.

**Fig. 2:**
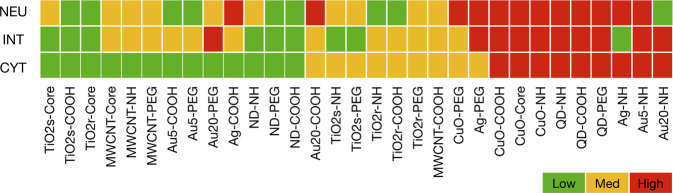
Classification tasks identified for the ENM safety classifier. Three different grouping approaches are proposed for ENM safety assessment. The 31 ENMs were first grouped on the basis of cytotoxicity data (CYT). Then, the second grouping of ENMs was defined based on an integration of genotoxicity, cytotoxicity, and immunotoxicity data using in vitro assays (designated as INT). Finally, the neutrophil count in BAL fluid was used to define the third categorization of ENMs reflective of their in vivo toxicity (NEU). Green represents a low hazard, yellow represents a medium hazard, and red represents a high hazard.

We then defined three different classification tasks (Fig. 2) based on cytotoxicity (CYT), integration of the in vitro assays (INT), and the in vivo neutrophil infiltration (NEU). In the CYT task, the 31 ENMs were grouped into the hazard categories according to their ability to cause similar patterns of cytotoxicity, while the INT task considers the combination of the homogenized assays by the means of the multi-view clustering algorithm Similar Network Fusion (SNF)^22^. In parallel, we profiled the immunotoxicity of the 31 ENMs in C57BL/6 mice, based on the total cell counts as well as the number of macrophages, neutrophils, eosinophils, and lymphocytes identified in the bronchoalveolar lavage (BAL) fluid. Neutrophil counts in BAL fluid after 4 days of exposure are a well-documented marker of acute inflammation in mice, as we and others have shown^23–26^. The neutrophils (NEU) counts were then used to divide the ENMs in the hazard categories: NoL (count <1), M (1 ≤ count <10), and H (count ≥10).

### Exploratory analysis based on PCA and univariate strategies

**Fig. 3:**
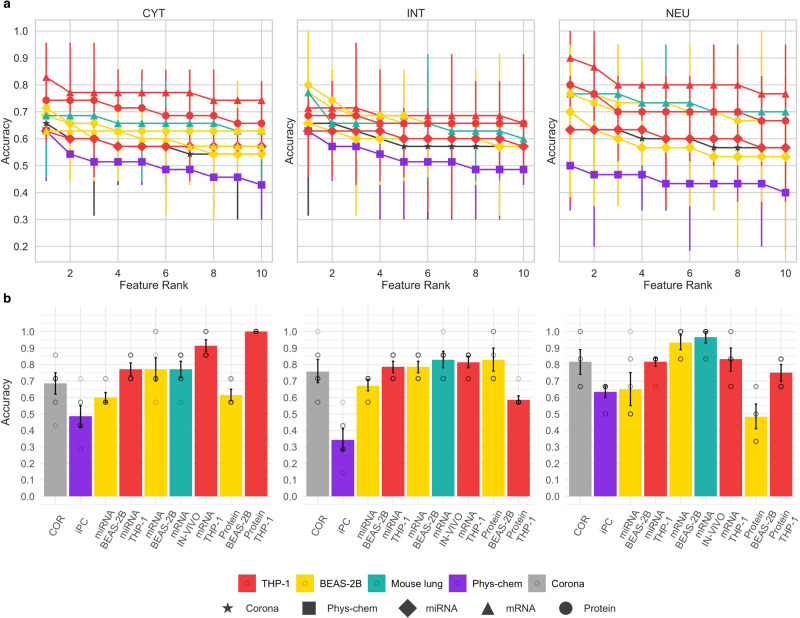
Comparison of selected models from different data layers and cell types. **a** Classification performances obtained from univariate-based models. Each panel reports the test set accuracy estimates (*n* = 5-fold cross-validation strategy). Data were represented as mean values and 95% confidence intervals. On the X-axis of each plot, the ten single top-performing features of each corresponding dataset grouped with respect to the toxicity labeling used are represented. **b** Classification performances obtained from multivariate-based models. Each panel reports the mean values of the test set accuracy estimates together with 95% confidence intervals (*n* = 10 best models). Colors indicate the cell model (THP-1 is represented in red, BEAS-2B is represented in yellow, and mouse lung is represented in turquoise), protein corona (represented in gray) and intrinsic properties (represented in violet and named as phys-chem), while the x-axis labels indicate the specific name of the employed data layer. The labels on the top indicate the classification tasks. (CYT) The testing accuracy of models selected for the cytotoxicity score, (INT) the integrated toxicity score, and (NEU) the in vivo toxicity-based classification task.

Next, we performed explorative analysis to assess the distributions of each data layer. For each dataset, the first two principal components were computed and used to plot the projected data points (Supplementary Figs. 2–4). Overall, this analysis highlights that different combinations of omics data layers and cell types convey distinct information about the mechanism of action of ENMs since the distributions of projected data points (ENMs) are remarkably different from each other. While the density of data points in the mRNA of the BEAS-2B dataset is the most homogeneous, the protein corona and all the datasets collected from THP-1 are heavily influenced by a few samples. Altogether, the scatterplots highlight the absence of linear separability of the toxicity classes by using all the available molecular features. This is not a surprising phenomenon in omics datasets, in which it is expected that the majority of the total variance of the data is associated with more than two dimensions^27^. Moreover, to define a baseline classification capability based on the sole predictive power of each independent variable in every dataset, the level of association between every variable in each dataset, and the toxicity class of the ENMs according to the different labeling schemes (CYT, INT, and NEU) was evaluated by fitting the univariate logistic regression model with repeated random splits (Fig. 3a). We found that single variables are only weak predictors of ENMs toxicity.

### Selecting multivariate biomarkers for ENM safety assessment

We further hypothesized that if any relationship between the intrinsic properties/molecular data and the toxicity grouping is present, it may be encoded in higher-order interaction terms. Therefore, we applied multivariate modeling strategies with the objective of finding compact sets of synergistic biomarkers.

We used toxicity-based groupings of the 31 ENMs as target *variables*, the omics datasets, and the intrinsic properties of the ENMs as *predictors*. Alternative feature selection and classification methods were used to determine the best composite biomarkers for each classification task (INT, CYT, and NEU). We applied standard machine learning (ML) techniques such as logistic regression with PCA (LR-PCA), LASSO (a regularization-based method) and varSelRF^28^, and GARBO^29^. GARBO is a specialized genetic algorithm that uses Fuzzy Logic and RF-based classifiers, to select biomarker sets that optimize the trade-off between classification accuracy and the number of biomarkers^29^. GARBO, LR-PCA, and LASSO were applied to select alternative biomarker models from the large-scale omics datasets (mRNA, miRNA, and proteomics). Compared to LR-PCA and LASSO methods, GARBO-generated composite biomarker models achieve the highest prediction accuracy in most of the used data layers (see Supplementary Figs. 5, 6). We systematically evaluated the classification performances of the selected biomarkers when using a different number of groups for each defined classification task (see Supplementary Fig. 7). Our evaluation highlights that the selected biomarker models achieve classification accuracies similar to those obtained when defining groupings of three classes. Figure 3 reports the classification performances of univariate models (Fig. 3a) and multivariate-based models selected by using the GARBO algorithm (Fig. 3b).

In the present paradigm, mRNA-, proteomics-, and protein corona-based models reached high accuracy (Fig. 3b). On the other hand, the models selected from the physicochemical properties performed less accurately as compared to the other data layers. As expected, the best models for the prediction of mouse lung neutrophil infiltration (NEU) were those built from the mRNA in vivo data. However, in the BEAS-2B cell line, the mRNA-based models also satisfactorily predict in vivo immunotoxicity (NEU), while proteomics-derived biomarkers have limited predictability both with respect to cytotoxicity (CYT) and immunotoxicity (NEU). On the other hand, protein-based feature sets in THP-1 cells exhibited high accuracy and stability scores for the cytotoxicity (CYT) classification task.

We also investigated whether the integration of different omics data types and physicochemical properties could improve the classification performance of the ENM safety classifier (Supplementary Fig. 8). We observed that ensemble classifiers derived from single or multiple data layers in THP-1 outperformed those derived from other biological models when considering the cytotoxicity classification task (CYT). However, when considering the integrated classification of toxicity (INT), the ensemble classifiers derived from single or multiple data layers in the BEAS-2B cell model systematically achieved the highest classification performances (Supplementary Fig. 8).

### Biomarkers to predict neutrophil infiltration

Bronchoalveolar lavage (BAL) immune cell identification and counting is a commonly accepted non-invasive procedure for the accurate and confident diagnosis of specific lung diseases^30^. Furthermore, neutrophil infiltration is a well-known marker of inflammation induced by ENM exposure. Here we asked whether the sets of specific biomarkers previously identified by means of linear regression would predict neutrophil infiltration. As shown in Supplementary Figs. 9, 10, all ten models based on the in vivo mRNA data displayed satisfactory performances (*R*^2^ > 0.6) with respect to neutrophil BAL counts in mice. Predicting in vivo endpoints from in vitro data to facilitate the implementation of the 3 R principles in nanosafety is currently a relevant topic. To this end, we analysed the transcriptomics generated in vitro to identify biomarkers that could serve as predictors of neutrophil infiltration in vivo (Supplementary Fig. 11 and Supplementary Table 12). Overall, all the models derived from BEAS-2B have good predictive performances (*R*^2^ > 0.6) and outperform the models generated from THP-1 data.

### External validation of mRNA-based classifiers

**Fig. 4:**
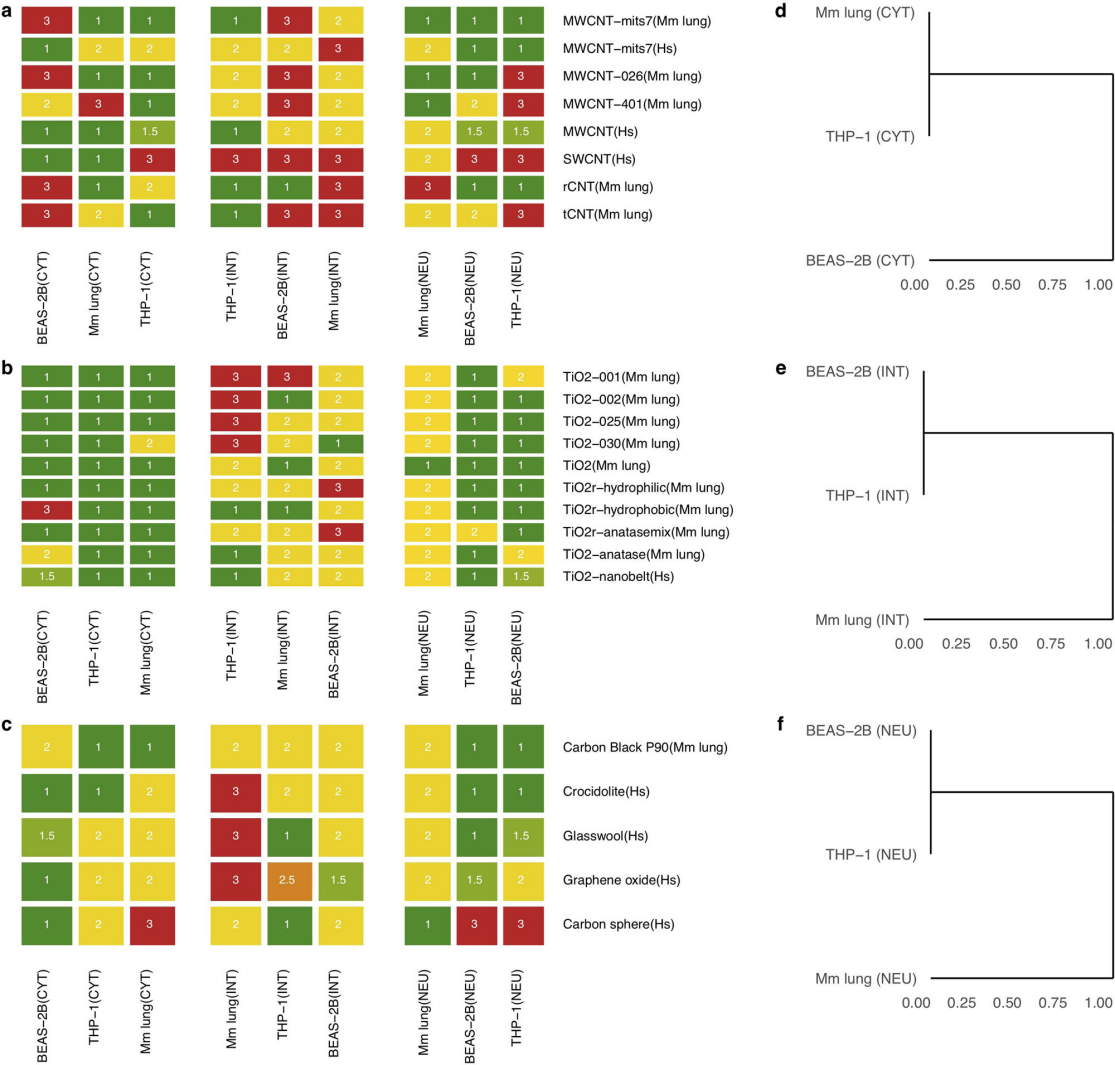
Prediction results on transcriptome profiles from external datasets. Heatmaps showing the class label assigned to each external ENM exposure, and dendrograms highlighting the distance between predictions made by using different cell models. **a** Prediction results on single- and multi-walled carbon nanotubes. **b** Prediction results on different TiO_2_ nanoparticles. **c** Prediction results on ENM types that were not included in the training set. **d** Dendrogram showing the distance between cell-based mRNA models selected for the classification task integrating different toxicity endpoints. **e** Dendrogram showing the distance between cell-based mRNA models selected for the cytotoxicity-related classification task. **f** Dendrogram showing the distance between cell-based mRNA models selected for the classification task defined on the basis of the neutrophil count in BAL fluid of mice. A color map was utilized to visually distinguish the predicted class labels: low (dark green), medium (yellow), and high (red) toxicity. In addition, since the predictions were summarized over the biological systems exposed to a given ENM, we reported the median value of these predictions and introduced two intermediate levels of toxicity: low to medium (light green) and medium to high (orange).

Next, external transcriptome datasets of in vitro and in vivo exposures to ENMs were retrieved from the NCBI GEO database (Supplementary Table 9) in order to validate the top ten mRNA-based biomarker sets selected for each exposure system (THP-1, BEAS-2B, and mouse lung) and classification task (CYT, INT, and NEU). Supplementary Tables 10–12 report the gene sets representing the best mRNA-based biomarker models. Some of the ENMs in the selected external datasets correspond to the same class of ENMs as the ones included in the training set (for instance, TiO_2_ and CNTs), while other types of materials were not represented in the training set (e.g., graphene oxide and crocidolite asbestos). For each biomarker set, an RF-based classifier was used to generate class probabilities. The scores from the top ten RF-based models were averaged to yield one set of class probabilities for each test (NoL%, M%, and H%), and the class associated with the highest score was chosen as the predicted class. Figure 4a–c reports the level of toxicity predicted by each model (cell type/classification task) on each external dataset. In order to improve the legibility of the evaluation results, the predictions derived from different doses of the same exposure were averaged. The top mRNA models indicated a high hazard priority for MWCNTs (Mitsui-7, or MWCNT-mits7 in Fig. 4). MWCNT-7 (Mitsui-7), a nanomaterial known to cause damage to the lungs^31^ and classified as a potential human carcinogen by IARC^32^. Crocidolite asbestos, a known carcinogen, was also predicted as hazardous by the models trained on the toxicity classes derived from the integration of different toxicity endpoints. Anatase TiO_2_ was predicted to be hazardous. Indeed, the anatase form of TiO_2_ is known to be chemically more reactive leading to greater toxicity in vitro and in vivo as compared to the rutile form^33,34^. TiO_2_-nanobelts were also predicted as highly hazardous and this is in line with the findings of the corresponding original study^35^, where the authors characterized patterns of gene expression in THP-1 cells and primary small airway epithelial cells exposed to high doses of TiO_2_ nanobelts. Figure 4d–f indicate how close the predictions are made by different cell type/mRNA models. It is interesting to observe that THP-1-derived predictive models are closer to in vivo models than those derived using BEAS-2B when focusing on cytotoxicity. This supports the hypothesis that differences in vitro models capture different aspects of the chemical exposures, hence collecting complementary data in multiple cell systems aids the in vitro-in vivo extrapolation of predictive biomarkers^36^. This is an important conclusion that accords well with previous studies aimed at assessing the capacity of in vitro assays to predict relevant in vivo outcomes^23^. Thus, it is unlikely that a single cell-based assay (focusing on a single endpoint) will accurately predict the more complex and concerted biological outcomes in vivo. Overall, the ENM safety classifier also yields robust and accurate results for external datasets and demonstrates the feasibility of a toxicogenomic-based safety classification of ENMs. Moreover, this is the first study in which predictive models of nanotoxicity are validated in a large collection of manually curated public datasets. Our analysis shows that, despite the profound differences in experimental design, material characterization, and omics technologies used, published data can be of considerable practical utility when properly curated and made available to the community.

### External validation of selected molecular markers

**Fig. 5:**
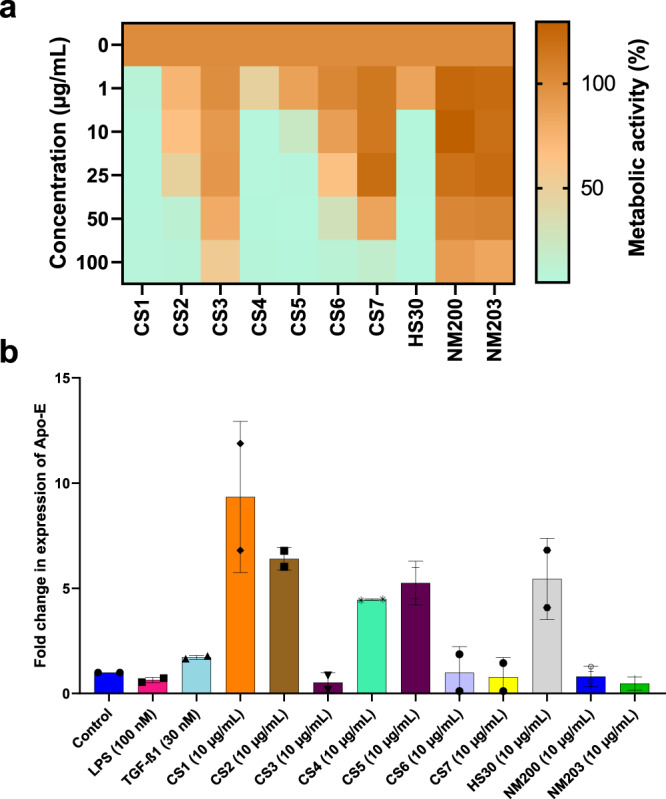
Molecular marker validation using the THP-1 model. Effect of a panel of silica ENMs on cell viability and expression of the *APOE* gene in THP-1 cells. **a** Heatmap showing changes in metabolic activity (corresponding to cell viability) of cells after 24 h of exposure to SiO2 ENMs. **b** Fold change in the expression of *APOE* mRNA at 24 h of exposure to 10 µg/mL. LPS (100 nM) and TGF-β (30 nM) were used as a reference. Data represent mean values ± SD (*n* = 2 independent experiments each performed in triplicate).

We observed that *APOE*, encoding apolipoprotein E, is consistently found in the THP-1-based models predictive of the cytotoxicity classification task (CYT) (Supplementary Table 10). To determine the validity of this molecular marker, we exposed THP-1 cells to a panel of amorphous SiO_2_ ENMs shown to display varying degrees of cytotoxicity (Fig. 5a). We then performed RT-PCR and found that the upregulation of *APOE* correlated with cytotoxicity when cells were exposed to 10 different SiO_2_ ENMs (Fig. 5b). This is thus fully in accordance with the CYT classifier for THP-1 cells (Supplementary Fig. 12). However, *SPNS2* was not validated as a biomarker of cytotoxicity.

### Biological roles of the selected biomarkers

Focusing on the mRNA-based classifiers, several genes of interest were identified from the Supplementary Tables 10–12. First, it is noteworthy that the BEAS-2B and THP-1 based models do not encompass the same genes nor do the in vitro models display similarities to the in vivo models at the level of the individual mRNAs. Second, for each model, it is noted that certain genes are more prevalent than others. For instance, *LDLR*, encoding the low-density lipoprotein receptor, is prominently featured in the case of the BEAS-2B-based models selected for the prediction of in vivo endpoints (NEU). Similarly, as noted above, *APOE*, encoding a lipid-binding protein, is consistently found in the THP-1-based models predictive of the cytotoxicity classification task (CYT) while *CHIL3*, encoding the chitinase-like 3 protein, is linked to the mouse lung-based models of in vivo classification. Despite the absence of endogenous chitin, a number of chitinases and chitinase-like proteins that bind but do not degrade chitin have been identified. These proteins play important roles in lung injury and are also known to play key roles in Th2-dominated disorders such as asthma^37,38^. In addition, a previous study using a mouse model of ovalbumin-induced asthma revealed that exposure to graphene oxide increased macrophage production of chitinases, CHI3L1, and AMCase^39^.

As stated above, all the THP-1 models derived from single or multiple data layers outperformed other biological models for integrated (INT) classification tasks. Interestingly, the THP-1-based model featured three genes, namely *AHRR*, *TMOD1*, and *NEAT1* (Supplementary Table 10 and Supplementary Fig. 13). *AHRR* encodes the aryl hydrocarbon receptor repressor (AhRR), which acts as a tumor suppressor gene in multiple human cancers^40^. Moreover, AhRR has a major impact on regulating inflammation^41^. *TMOD1* encodes the tropomodulin 1 protein (Tmod1), whose role in nanotoxicity is unexplored, but it could be linked to actin cytoskeleton-related responses to ENMs. In fact, SWCNTs were previously shown to reorganize cellular actin structures^42^. *NEAT1* (nuclear paraspeckle assembly transcript 1), in turn, is a long non-coding RNA (lncRNA) known to be upregulated in multiple malignancies^43^. Recently, *NEAT1*, a target gene of the tumor suppressor gene p53, was shown to enable tumorigenesis in vivo by promoting the survival of oncogene-targeted cells^44^. Interestingly, extracellular vesicles enriched in lncRNAs, such as NEAT1, drive fibrosis in a mouse model of ischemic heart disease^45^. NEAT1 has also been suggested to drive the progression of liver fibrosis^46^ and, more recently, its role in the promotion of pulmonary fibrosis has been shown^47,48^. Indeed, fibrosis is a commonly observed adverse outcome related to ENM exposures, especially in the lung^49,50^. This supports our hypotheses that integrated methods may be applied to identify early biomarkers predictive of long-term outcomes of exposure. Altogether, these results not only provide a means to establish innovative tools for the prediction of the toxicity of ENMs but also clarify important aspects related to the intermediate mechanisms of the exposure. This information can be used in further dedicated studies to draft new adverse outcome pathways and refine existing ones.

## Discussion

The chemical industry is undergoing a profound reorientation of research and development towards “safe-and-sustainable-by-design” according to the principles of green chemistry. To fully unleash the power of this new paradigm, the nanotechnology industry needs to gain full access to integrated models that take into consideration both intrinsic and biological properties of the ENMs. These models go beyond the traditional predictive models in which the exposure is linked to an apical endpoint, by adding key elements of the mechanisms underpinning the biological responses. This is the first study in which such an approach has been used to analyze a large collection of industrially relevant ENMs with unprecedented depth. It should still be pointed out that these integrated approaches do not replace traditional toxicity testing or QSAR modeling. Instead, they provide a unique complementary view of the chemical–biological interactions. The work presented here is novel and ground-breaking for the present and future nanosafety for multiple reasons. Our work presents the largest in-depth characterization of intrinsic and biological properties for a selection of 31 industrially relevant nanomaterials. However, certain common ENMs were not included in the panel, due to limited resources, and for reasons having to do with the amenability of some ENMs to surface modification. Nonetheless, the dataset was designed to be as representative as possible, and the PCR validation of the identified biomarkers on an external set of silica ENMs (not included in the original panel) was found to correlate with cytotoxicity. This suggests that the applicability of the identified biomarkers robustly extends beyond the set of ENMs used for their discovery. Moreover, we showcase an integrated modeling approach to define hybrid predictive models of toxicity, comprising both intrinsic and mechanistic properties of ENMs. The best classification performances were obtained with multiple classifier systems integrating the model predictions of RF-based classifiers trained on biomarker sets selected from different omics data types. In our paradigm, predictive models relying exclusively on physicochemical properties of the ENMs achieved lower accuracy. However, their accuracy was improved by building hybrid models in which intrinsic ENM properties and omics-driven mechanism of action information was combined. Although carefully selected, the ENMs investigated here do not cover the full spectrum of nanomaterials presently available on the market. However, our validation results and the full availability and reusability of our data/source code in accordance with the FAIR principles^51^ will allow expanding and refining of our models as new data will become available. Furthermore, the most accurate models were validated in a large selection of manually curated toxicogenomics datasets as well as newly generated molecular data. In addition, our results highlight new biomarkers of toxicity that anchor the toxicity potential of ENMs to specific molecular and cellular functions, thus facilitating the generation and refinement of ENM-specific AOPs. Finally, we prove that profiling the molecular alterations of biological systems after ENM exposure at subtoxic doses, provides not only molecular proxies of toxicity but also knowledge of the mechanism of action of the exposure. In sum, the integrated models presented here predict the hazard potential of ENMs and may guide the prioritization of ENMs.

## Methods

### Synthesis and characterization of ENMs

Details on the synthesis and characterization of the 31 ENMs studied herein along with further details on the experimental protocols used to generate the in vitro and in vivo toxicity and omics data, and methods deployed for the validation of selected biomarkers, are found in the Supporting Information file (and refer to ref. ^14^ for ENMs).

### Computational infrastructure

Data on ENM physicochemical properties, in vitro and in vivo toxicity, and different omics datasets, as described above, were derived from exposures to 31 different ENMs. This large set of data were exploited to identify biomarkers of ENM toxicity, to build predictive classifiers, and to validate the RF-based models on other ENM exposures obtained from external datasets. This was achieved through the implementation of a computational infrastructure consisting of the following steps: (1) identification of targeted classification tasks of toxicity, (2) selection of relevant intrinsic and biological properties enabling toxicity assessment of ENMs, (3) comparison of selected models with simpler computational methods (GARBO vs LASSO and LR-PCA), (4) testing in vivo based mRNA biomarkers for the prediction of neutrophil count by using a regression-based model, (5) evaluation of ensemble models integrating more than one single-view classifier, and (6) validation by using external datasets (focusing on transcriptomics data due to their greater availability as compared to other omics data).

### Grouping of the studied ENMs

Toxicity data generated from high-throughput screening methods were employed to identify shared patterns of toxicity across the selected 31 ENMs. We identified three different groupings of the 31 ENMs, one based on an integration of cyto-, geno-, and immunotoxicity data using in vitro models (INT—in vitro), one based solely on cytotoxicity data (CYT—in vitro) and one based on neutrophil counts in BAL fluid (NEU—in vivo). Specifically, we used toxicity assays to measure DNA and chromosome damage in the BEAS-2B cell line (genotoxicity), cell viability (cytotoxicity) in multiple different cell types (BEAS-2B, Jurkat, THP-1), and cytokine profiles for THP-1 cells (immunotoxicity). We decided to conduct a separate classification analysis for the cytotoxicity data since they rely on different cell models and show more consistent toxicity profiles across the 31 ENMs. The in vitro cytotoxicity and genotoxicity assay data were homogenized by applying a point-based classification system. Given a toxicity endpoint, this system assigns a number, ranging between 1 (no toxicity) and 6 (high toxicity), indicating a general toxicity score. The categorization-based system along with the conversion results are reported in the Suppl. Information file. After homogenizing the toxicity assay data, (i) the k-means algorithm implemented in R was employed to create groups of ENMs based on their cytotoxicity profiles, here indicated as CYT (more details in next section). Then, a multi-view clustering algorithm, namely similar network fusion (SNF), was used to create a grouping of ENMs based on the integration of their cyto-, geno-, and immunotoxicity profiles, here indicated as INT. Finally, we defined the third grouping of ENMs based on the BAL neutrophil levels, here indicated as “NEU” (for neutrophils). In particular, the neutrophil counts were exploited to define a third grouping of the ENMs which consists of the following three categories: NoL (value <1), M (1 ≤ value <10), and H (value ≥10).

### Assessing the number of clusters for grouping

We assessed how data would group according to the toxicity assay data. To this end, for each toxicity score, we performed a cluster analysis by fitting a gaussian mixture model (GMM) with a spherical covariance matrix for each cluster. We compared the results by varying the number of clusters, ranging from 2 to 6 using the BIC. The BIC can be interpreted as a goodness-of-fit score that penalizes overly complex models; the lower the score, the better the fit. For each number of clusters, we fitted the GMM 250 times to evaluate the influence of the random initialization on the algorithm and compared the respective distributions of the BICs. A larger variance of BICs indicates that the model depends heavily on the random initialization (this is evident for the case of 5 and 6 clusters, where the chance of overfitting is higher and represented by the many individual models with BIC values very distant from the average). On the other hand, when the number of clusters is kept between 2 and 4 the values of the BIC are compactly distributed around their respective mean values, implying that the clustering algorithm is not so influenced by the random initialization. Here, the results are reported by dividing the ENMs into three groups. This value was chosen as a trade-off between data variability and the stability of the results. For comparison, the same results are reported for different, equally reasonable, groupings k = 2, 4 in Supplementary Fig. 1.

### Training, validation, and testing of RF-based classifiers

In order to address the feature and classification tasks, we partitioned the datasets into training (70%) and testing (30%). Because of the relatively limited number of studied ENMs, the training test was utilized for both model selection and training of RF-based classifiers on the final selected models. The implemented ML-based evaluation strategy defined training, validation, and testing sets. The classification accuracy was systematically compiled on the test sets (30%), while the training sets (70%) were used to select the biomarker models (or feature subsets) and to train RF-based classifiers. The initial splitting in the training and testing set is repeated five times. Then, GARBO utilizes k-fold cross-validation for model evaluation and selection. Note that the univariate analysis is also based on the defined training and test sets.

### Exploratory analysis based on PCA and univariate strategies

Prior to PCA analysis, each layer of omics data has been standardized in order for each variable to have an average value of 0 and a standard deviation of 1. After standardization, each dataset was projected on its corresponding first two principal components and plotted for graphical inspection. On each training set (70%), each variable was first standardized, then a univariate logistic regression was fitted and evaluated on the corresponding test set. For each variable in each dataset, the test set (30%) performances were collected at each split. The best-ranking features were chosen according to the best mean test set accuracies achieved and reported in Fig. 3a together with their corresponding 95% confidence interval as a measure of the spread of the estimate.

### Marker selection for the prediction of toxicity

While toxicity assay data were used to define different categorizations of the ENMs, omics profiling and the intrinsic properties of ENMs were employed to identify predictive markers of ENM-induced toxicity. Given one categorization of ENMs, different feature selection and classification algorithms were used. In particular, we tested two standard methods such as logistic regression combined with principal component analysis (LR-PCA) and LASSO. LASSO is a regression analysis method that performs both variable selection and regularization in order to enhance the prediction accuracy of the final trained models. Both models LR-PCA and LASSO were trained using the defined five-repeated train/test splits, with proportions of 70/30%. In both settings, each variable in the training set has been standardized to have a mean of 0 and a standard deviation of 1 (the learned standardization is equally applied to the corresponding test split). For the LR-PCA model, due to data availability, only the first two principal components were systematically used to fit the logistic regression model. For the LASSO model, a further model selection step on the training split has been performed to find the best regularization parameter. In particular, the training set of each split is further divided into a training set and a validation set according to a fivefold cross-validation scheme. The nested cross-validation scheme was used to estimate the performance of different models indexed by the regularization parameter which varied in the range [2^−3^, 2^−1^]. The best parsimonious model was selected using the “one standard deviation” empirical rule^52^. After identification of the best parameter, a new model is again fitted on the whole training set this time and the generalization capabilities were evaluated on the corresponding test set. For both LR-PCA and LASSO models, the test set performances across the five splits were collected and aggregated into a mean value and a 95% confidence interval and reported in Supplementary Figs. 5, 6. Then, we applied the GARBO feature selection algorithm which enables the selection of very small and highly accurate biomarker models from large-scale genomics data. More detailed information about GARBO can be found in ref. ^29^. LR-PCA, LASSO, and GARBO were used to select multiple informative marker sets from the mRNA, miRNA, and proteomics data layers. The backward variable elimination algorithm, namely varSelRF^28^, was used to select feature sets from the intrinsic properties and protein corona profiles. Classification performances were calculated on the testing sets and by using the overall classification accuracy. We used the overall classification accuracy as the main metric for the evaluation of biomarker models, because of the limited number of samples in the testing sets (average of 6). Test accuracy and stability-based metrics were used to select the top ten mRNA-based biomarker models for the external validation tests and the discussion on the most interesting biomarkers. The stability was calculated with the Dice–Sorensen’s index, which is always in the range of [0, 1]. Stability aims at measuring the capability of the feature selection process in reproducing (more or less) the same feature subsets with different training sample sets. High stability is often correlated to high reproducibility. The classification accuracy and the Dice score were finally used to rank the multiple marker sets (or markers of toxicity) generated by the feature selection and classification algorithms. Specifically, the rank was compiled as the weighted sum of the classification accuracy (*w* = 0.5) and the Dice stability score (*w* = 0.5).

### Regression-based models for predicting BAL cell counts

The mRNA markers identified by means of the genetic algorithm on the in vivo and in vitro mRNA datasets for the in vivo classification tasks were used to build a linear regression model to predict the neutrophil BAL cell counts. The linear regression modeling was performed by means of the lm function from the stats R library. Before modeling, the neutrophil cell counts were log-transformed.

### External datasets focusing on mRNA-based features

The RF-based classifiers built upon the top ten mRNA marker sets selected for each classification task were validated with mRNA profiles derived from external ENM-related studies. The mRNA-based models were selected based on a weighted sum between test accuracy and the stability calculated with the Dice coefficient. Supplementary Table 9 reports Gene Expression Omnibus (GEO) ID and a brief description of the selected external (publicly available) ENM studies. The prediction of the class labels for each test was made upon the class probabilities (NoL%, M%, and H%) generated by the top ten RF-based models. The scores from the top ten RF-based models were finally averaged to yield one set of class probabilities for each test, and the class associated with the highest score was chosen as the predicted class label.

### Molecular marker validation by RT-PCR

To attempt the validation of selected molecular markers, we used a non-related set of amorphous SiO_2_ ENMs of different sizes (CS1, CS4, CS5, CS6, and CS7) and surface modifications (CS2 and CS3). These colloidal silica (CS) particles were obtained from Nouryon (formerly Akzo Nobel Pulp and Performance Chemicals AB) (Bohus, Sweden) and the properties of these materials were previously reported^10^. In addition, Ludox HS30 (Sigma-Aldrich) and two additional amorphous silica nanoparticles (NM200 and NM203) obtained from the Nanomaterial Repository of the Joint Research Centre (JRC) were included. The properties of the latter materials were described in refs. ^11,12^, respectively. The dispersions of CS NPs and Ludox HS30 were prepared by dilution to working concentrations in a cell culture medium, while dispersions of NM200 and NM203 were prepared by probe sonication as reported previously in FP7-NANOREG^12^.

#### Cytotoxicity assay

The human monocyte-like cell line THP-1 was grown in RPMI-1640 medium supplemented with 10% heat-inactivated FBS (Sigma), 2 mM glutamine (Gibco), penicillin (100 U/mL), and streptomycin (100 µg/mL). THP-1 cells were exposed to silica ENMs (1–100 µg/mL) and the loss of cell viability was determined using the Alamar blue assay which is based on the metabolic conversion of resazurin, a nonfluorescent indicator dye, to red-fluorescent resorufin in living cells^12^ (Thermo Fisher Scientific, Sweden). The results derived from three independent experiments each performed in triplicate are presented as a heatmap depicting low (green), medium (yellow), or high (red) toxicity.

#### RT-PCR

Cells were seeded in six‐well plates and exposed to a panel of SiO_2_ NPs at 1 and 10 µg/mL for 24 h. After exposure, cells were collected and washed with PBS before processing for RNA isolation. RNA was isolated using the QIAGEN RNeasy Mini Kit by following the manufacturer’s protocol. The quality and yield of RNA was checked using NanoDrop (ThermoScientific). cDNA was synthesized using iScript^TM^ Reverse Transcriptase Kit (Bio‐Rad) using a thermal cycler (Bio‐Rad). RT‐PCR was performed using SYBR‐Green-based 96‐well primePCR custom plates (Bio‐Rad) for the following genes: *APOE (qHsaCED0044297)*, *SPNS2 (qHsaCID0008369)*, and *GAPDH* (qHsaCED0038674). Each RT‐PCR reaction contained 1 µL of cDNA, 1x SsoAdvanced universal SYBR supermix (Bio‐Rad), and 1x PrimePCR assay dried in a well. RT‐PCR was run using the AB7500-Standard RT‐PCR (Applied Biosystems) at the following conditions: activation at 95 °C for 2 min, 40 cycles of denaturation at 95 °C for 5 s, and annealing/elongation at 60 °C for 30 s. The fold change in the gene expression was obtained by calculating the ΔΔCt value with respect to *GAPDH* as reference.

### Reporting summary

Further information on research design is available in the Nature Research Reporting Summary linked to this article.

## Supplementary information

Supplementary Information

Reporting Summary

## Data Availability

The processed mRNA, miRNA, proteomics, protein corona, physicochemical properties, and BAL cell counts used in this paper have been deposited in the online Zenodo repository under the accession number 10.5281/zenodo.4247173. Data were also available from the corresponding authors upon request.

## Code availability

The scripts used to perform the described analyses, are available from the online repository Zenodo (10.5281/zenodo.4247173).
