# Supplementary material for: Biomarkers of nanomaterials hazard from multi-layer data
Source: Nat Commun. 2022 Jul 1;13:3798. doi: 10.1038/s41467-022-31609-5 (PMC9249793; doi:10.1038/s41467-022-31609-5)
Supplement: Supplementary file 1 — Supplementary Information [file 41467_2022_31609_MOESM1_ESM.docx]

Supplementary Information

**Biomarkers of nanomaterials hazard from multi-layer data**

Vittorio Fortino^1^, Pia Anneli Sofia Kinaret^2,3,4,5^, Michele Fratello^2,3,5^, Angela Serra^2,3,5^, Laura Aliisa Saarimäki^2,3,5^, Audrey Gallud^6^, Govind Gupta^6^, Gerard Vales^7^, Manuel Correia^8^, Omid Rasool^9^, Jimmy Ytterberg^10^, Marco Monopoli^11^, Tiina Skoog^12^, Peter Ritchie^13^, Sergio Moya^14^, Socorro Vázquez-Campos^15^, Richard Handy^16^, Roland Grafström^6,17^, Lang Tran^13^, Roman Zubarev^10^, Riitta Lahesmaa^9^, Kenneth Dawson^18^, Katrin Loeschner^8^, Erik Husfeldt Larsen^8^, Fritz Krombach^19^, Hannu Norppa^7^, Juha Kere^12^, Kai Savolainen^7^, Harri Alenius^6,20^, Bengt Fadeel^6^, and Dario Greco^2,3,4,5,*^

*^1^ Institute of Biomedicine, University of Eastern Finland, Kuopio, Finland;*

*^2^ Faculty of Medicine and Health Technology, Tampere University, Tampere, Finland;*

*^3^ BioMediTech Institute, Tampere University, Tampere, Finland;*

*^4^ Institute of Biotechnology, University of Helsinki, Helsinki, Finland;*

*^5^ Finnish Hub for Development and Validation of Integrated Approaches (FHAIVE)*

*^6^ Institute of Environmental Medicine, Karolinska Institutet, Stockholm, Sweden;*

*^7^ Finnish Institute of Occupational Health, Helsinki, Finland;*

*^8^ National Food Institute, Technical University of Denmark, Kgs. Lynby, Denmark;*

*^9^Turku Bioscience Centre, University of Turku, and Åbo Akademi University, Turku, Finland;*

*^10^Department of Medical Biochemistry and Biophysics, Karolinska Institutet, Stockholm, Sweden;*

*^11^Department of Pharmaceutical and Medicinal Chemistry, Royal College of Surgeons in Ireland, Dublin, Ireland;*

*^12^ Department of Biosciences and Nutrition, Karolinska Institutet, Huddinge, Sweden;*

*^13^Institute of Occupational Medicine, Edinburgh, UK;*

*^14^Soft Matter Nanotechnology Laboratory, CIC biomaGUNE, San Sebastian, Spain;*

*^15^Leitat Technological Center, Terrassa, Spain;*

*^16^School of Biological and Marine Sciences, University of Plymouth, Plymouth, UK;*

*^17^Division of Toxicology, Misvik Biology, Turku, Finland;*

*^18^Centre for BioNano Interactions, School of Chemistry and Chemical Biology, University College Dublin, Dublin, Ireland;*

*^19^Walter Brendel Centre of Experimental Medicine, Ludwig-Maximilians-Universität München, Munich, Germany;*

*^20^Department of Bacteriology and Immunology, University of Helsinki, Helsinki, Finland.*

*To whom correspondence should be addressed. E-mail: [dario.greco@tuni.fi](mailto:dario.greco@tuni.fi)

**SUPPLEMENTARY INFORMATION FILE CONTENT:**

**Expanded Methods**

**Suppl. Results and Discussion**

**Suppl. References**

**Suppl. Figures S1-S13.**

**Suppl. Tables S1-S12.**

### EXPANDED METHODS

#### ENM synthesis

The NANOSOLUTIONS consortium prepared ENMs (Table 1) with three types of surface functionalization: carboxyl/carboxylate groups (COOH/COO^-^), amino/ammonium groups (-NH_2_/-NH^3+^) or polyethylene glycol (PEG). Additionally, in the case of TiO_2_ spheres and rods, CuO particles and MWCNTs, the non-functionalized variants designated as “core” ENMs were synthesized. Below follows a brief description of the synthesis of the ENMs (nominal size or aspect ratio and form are given in parentheses).

Table 1: The set of 31 ENMs.


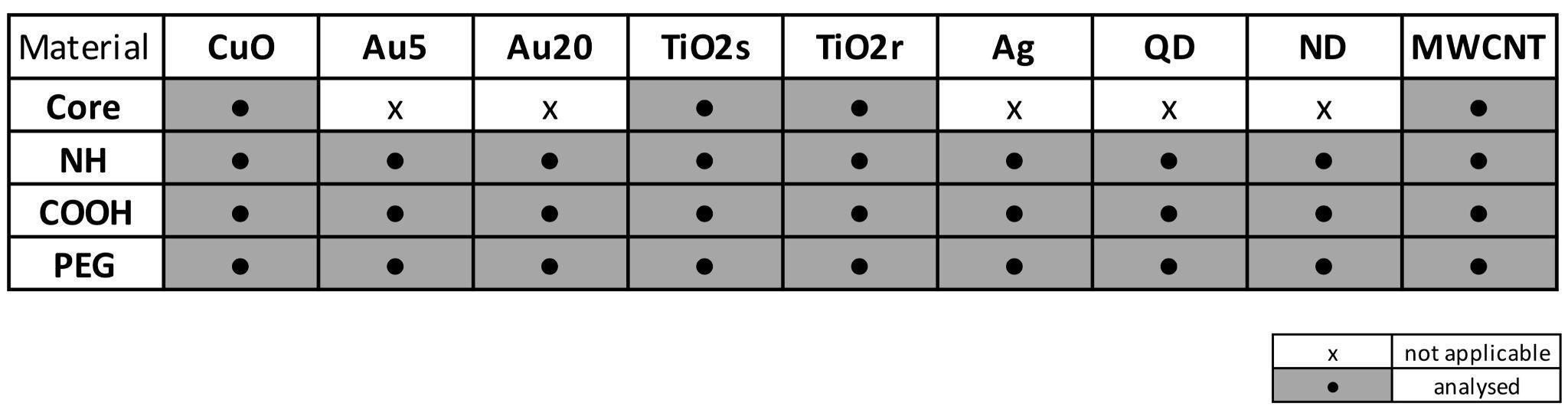


*TiO_2_ particles (10-20 nm, powder) and TiO_2_ rods (1:5 aspect ratio, powder – exception: TiO2s-PEG concentrated suspension)*: Colloidal solutions of nano-sized TiO_2_ were prepared at room temperature by hydrolysis of titanium tetrachloride solution with a further condensation of the reaction products, and stabilization by nitric acid. For the synthesis of TiO_2_ nanorods, different conditions were tested: forced hydrolysis in acidic conditions at normal pressure; forced hydrolysis at high pressure at different temperatures in alkaline conditions. The first synthesis approach had the highest yield and allowed the production of nanorods with the desired diameter and aspect ratio. The resulting materials were dried under vacuum at mild temperatures. For surface modification of TiO_2_ spheres and rods, a multistep procedure was developed. First, the particles were modified with a NH_2_-terminated silane. This yielded amino-modified TiO_2_. As a next step, to produce –COOH and –PEG modified particles, succinic anhydride and PEG-COOH, respectively, were bound to the amino group through an amide bond.

*CuO particles (10-20 nm, powder – exception: Cu-PEG concentrated suspension):* CuO nanoparticles were synthesized by the precipitate decomposition method. A precursor, basic copper carbonate (Cu_2_(OH)_2_CO_3_), was first prepared by precipitation reaction from an aqueous solution composed of 1 M copper nitrate and sodium carbonate. The obtained precursor was dewatered, dried and milled. Nanocrystalline CuO particles were obtained by thermal decomposition of the precursor at 300°C for 2 h. For surface modification of the CuO particles, CuO nanopowder was milled with 0.5 mm zirconia beads in a solution of a ligand (with thiol groups) in ethanol during 1 h at 2570 rpm (estimated speed of zirconia beads 19 m/s). Then the zirconia beads were separated by decantation and the modified particles purified by repeated precipitation and re-dispersion in ethanol. The following ligands were used: -HS-CH_2_-COOH (CuO-COOH), -HS-(CH2)_2_-NH_3_Cl (CuO-NH_2_) and –HS-CH_2_-COO-PEG(550)OCH_3_ (CuO-PEG).

*Au particles “Au5” (3-6 nm, suspension):* Gold nanoparticles with a core size < 5 nm were prepared using reduction of Au^3+^ to Au^0^ with sodium borohydride NaBH_4_ in the presence of bi-functional ligands of the type XRSH (X = COOH, N(CH_3_)_3_ or CH_3_) so that the surface was terminated with these functionalities. The ligands bound to the gold surface through their thiolate (RS) end. For carboxylate functionalization, the bi-functional ligand 11-mercaptoundecanoic acid was mixed with Au^3+^. Excess surfactants and other ionic by-products were removed by dialysis. Au-5-ammonium was synthesized *via* Murray-type place displacement of 2 nm n-dodecyl mercaptan-functionalized gold nanoparticles with N,N,Ntrimethyl(11-mercaptoundecyl) ammonium bromide units. The nanoparticles were purified by removal of solvent under vacuum, and by repeated washing with dichloromethane. Au-5-PEG was prepared by direct synthesis with a controlled ratio of HAuCl_4_ / PEG methyl ether thiol (average 550 g/mol). Au-5 ENM were purified by dialysis from aqueous nanoparticle suspensions in pure water over a 7-day period. For further details, see ref. 1.

*Au particles “Au20” (10-20 nm)*: Gold nanoparticles with a core size > 10 nm were synthesized by reduction of Au^3+^ to Au^0^ with sodium citrate. The citrate reduction process involved hot gold chloride and sodium citrate as reactants. In this reaction, the citrate molecules act as both reducing and stabilizing agents, allowing for the formation of the colloidal gold. The bi-functional ligands of the type XRSH (X = COOH, N(CH_3_)_3_, and CH_3_) were used in order to replace the citrate ligands on the nanoparticles surface. Au-20-carboxylate were obtained by exchange of citrate molecules with the thiol ligand HS(CH_2_)_10_CH_2_O(C_2_H_4_O)_3_C_2_H_4_OCH_2_CO_2_H. For Au-20-ammonium, *N*,*N*,*N-*trimethyl(11-mercaptoundecyl)ammonium bromide was used to exchange citrate ligands. For Au-20-PEG particles, PEG methyl ether thiol (average 550 g/mol) was the bi-functional ligand used in the synthesis. The Au-20 nanoparticles were purified by dialysis from aqueous nanoparticle suspensions in pure water over a 7-day period.

*Ag particles (10-20 nm, suspension):* Ag nanoparticles were synthesized by reduction of Ag^+^ to Ag^0^ with NaBH_4_. The bi-functional ligands of the type XRSH (X = COOH, N(CH_3_)_3_ and CH_3_) were added and also provided an efficient stabilization. For Ag-carboxylate, 11-mercaptoundecanoic acid was mixed with a silver nitrate solution in THF/H_2_O. Use of THF/H_2_O (5:1) as solvent provided the right environment to allow the initial solubilization of silver nitrate and the fast precipitation of 11-mercaptoundecanoic-coated nanoparticles after reduction. After the annealing of the Ag nanoparticles, the population of small particles (clusters) practically disappeared. The UV/VIS-absorption band experienced a blue shift and became narrower with the increase in the annealing time. For Ag-ammonium, the polar N,N,N-trimethyl-(11-mercaptoundecyl)-ammonium bromide ligand was added followed by addition of NaBH_4_. Ag-PEG was synthesized by reduction of Ag^1+^ to Ag^0^ with NaBH_4_ during 8 h. PEG methyl ether thiol (average 550g/mol) ligand**s** were added to provide stabilization. The Ag nanoparticles were purified by dialysis from aqueous nanoparticle suspensions.

*CdTe particles/quantum dots (3-5 nm, powder):* CdTe QDs were synthesized by a proprietary modification of the procedure described in the literature (PlasmaChem GmbH). Cadmium and tellurium precursors were mixed together with a thiocarboxylic acid, which served as a stabilizing ligand. After introduction of the reducing atmosphere, the solution was refluxed until a desirable wavelength of emission (approximately 590 nm) was achieved. Upon reaching the desirable emission wavelength, QDs were precipitated by isopropanol. Several washing and centrifugation cycles (300 g) were followed by drying at 50°C under vacuum to yield a dry powder. Surface modification of the CdTe QDs with carboxyl-, amino- and PEG groups was performed through ligand exchange with the respective thiols (mercaptocarboxylic acid for CdTe-COOH).

*Nanodiamond particles (3-5 nm, powder):* First, ND soot was produced by explosion of mixed trinitrotoluene-hexogen (TNT-RDX 60/40) in a large explosion chamber (50 m^3^) at a raised pressure of a cooling gas. Then, metals and non-diamond carbon were oxidized with a HNO_3_-HClO_4_ mixture and the NDs were isolated. NDs containing 0.09 mmol/g of carboxylic groups were used for further chemical modification in order to synthesize the amino- and PEG-modified NDs by amide and ester formation, respectively.

*Multi-walled carbon nanotubes (1:100 aspect ratio, powder)*: MWCNTs were produced through a catalytic chemical vapor deposition (CCVD) by decomposing a hydrocarbon source on a catalyst under appropriate operating conditions (*e.g.*, temperature, gas flow, time) using patented technologies for catalyst and process (Nanocyl NV). The MWCNTs consisted of around 10 walls. Purification was performed to diminish remaining catalyst residual content. Purification mainly consisted in treating the MWCNTs in acid oxidative solution to solubilize the different metallic structures. After purification, intense washing with water was performed to obtain neutral pH. -COOH surface functionalized MWCNTs were obtained from purified CNTs *via* an oxidative procedure. The –NH_2_ functionalized MWCNTs were produced from purified MWCNTs *via* ball-milling under reactive atmosphere (NH_3_) (patent WO 2002/0220402 A1). To generate MWCNT-PEG, a non-covalent functionalization process was applied. 125 mg PEGylated lipid, 1,2-distearoyl-sn-glycero-3-phosphoethanolamine-N-[methoxy(polyethylene glycol)-2000], DSPE-PEG_2000_ was first dissolved in 50 mL of distilled water and then added to the pristine MWCNT (25 mg) at a 1:5 MWCNT:DSPE-PEG_2000_ weight ratio. The mixture was sonicated at room temperature for 1 h and diluted to 250 mL with water before filtration through a 100 kDa cut-off filter (Millipore) to remove any unbound DSPE-PEG_2000_. The dilution/filtration step was repeated 5 times. The final product was resuspended in water to give the final product of MWCNT-PEG (NSNP704) at 1:0.65 MWCNT:DSPE-PEG_2000_ weight ratio.

#### ENM characterization

ENMs were characterized using a variety of analytical techniques and details are provided in Gallud et al.^1^ In brief, transmission electron microscopy (TEM) was applied to determine particle primary size, aspect ratio (for non-spherical ENM) and to qualitatively evaluate agglomeration/aggregation state (presence of agglomerates/aggregates yes/no). Dynamic light scattering (DLS) was used to measure hydrodynamic size (Z_ave_) and polydispersity index (PDI) for some of the ENMs (TiO_2_, CuO, Ag) and laser Doppler microelectrophoresis to determine Zeta potential. The medium for the latter was ultrapure water or 10 mM NaCl. Hydrodynamic diameters were further determined by differential centrifugal sedimentation (DCS). Brunauer–Emmett–Teller (BET) surface area analysis was applied to determine the specific surface area for ENM in the powder form. It was not possible to obtain specific surface area values for the various CdTe QDs, TiO_2_-ammonium, TiO_2_-carboxylate, TiO_2_-PEG, and CuO-PEG as these ENMs contained a large quantity of ligands, which resulted in anomalous adsorption/desorption isotherms. The presence of impurities (Al, Si, Mn, Fe, Co, Ni, Cu, Cr, Ga, Fe, Br, Sr, As, Mo, Ag, Sn, Sb, Te, W, Au, Pb) was investigated by inductively coupled plasma-mass spectrometry (ICP-MS). The release of ions from metal or metal oxide ENMs was determined by ICP-MS after dialysis in ultrapure water for 24 h. The presence of different functional groups was verified with X-ray photoelectron spectroscopy (XPS), Fourier transform infrared spectroscopy (FTIR), and thermogravimetric analysis (TGA). Detailed results are reported in ^1^.

#### Cell culture and cytotoxicity evaluation

The human monocytic THP-1 cell line was purchased from the American Type Culture Collection. The cells were cultured in RPMI-1640 medium supplemented with 10% fetal bovine serum (FBS), 2 mM glutamine, 100 U/mL penicillin, 100 μg/mL streptomycin, and 0.05 mM β-mercaptoethanol. The cells were not differentiated into macrophage-like cells. The transformed (non-malignant) human bronchial epithelial cells BEAS-2B were obtained from the American Type Culture Collection. The cells were grown in serum-free BEGM medium at 37°C in a humidified atmosphere of 5% CO_2_. Cytotoxicity towards THP-1 cells was determined by using the Alamar Blue cell viability assay (Thermo Fischer) as described.^2^ To this end, THP-1 cells were seeded into a 96-well plate at a density of 1 x 10^6^ cells/mL. After exposure to ENMs, the Alamar Blue reagent was added for 4 h at 37°C. The resulting fluorescence was determined at 540/590 nm (ex/em), using an Infinite 200 Tecan microplate reader operating with Magellan v7.2 software. Cytotoxicity toward BEAS-2B cells was measured using the Trypan Blue dye exclusion technique (after collecting cells by trypsinization) by counting the number of living (unstained) cells using a phase-contrast microscopy.^3^

#### Extraction of RNA/miRNA and DNA

Cells were exposed to the EC_10_ dose (for the cytotoxic ENMs) or a fixed dose for non-cytotoxic ENMs (100 µg/mL for THP-1 cells, and 200 µg/mL for BEAS-2B cells). Cell culture medium without ENMs was employed as negative control. Cells were then harvested and washed twice with ice-cold PBS (directly on the plate for the adherent BEAS-2B cells or by centrifugation 300g, 5min, 4°C for the THP-1 cells grown in suspension) and extraction of samples was performed as described.^4^ The concentrations of RNA and DNA concentrations were measured using a Nanodrop spectrophotometer. RNA and DNA samples were stored at -80℃ and -20℃, respectively, prior to analysis.

#### Mouse exposures to ENMs and RNA extraction

Female C57BL/6 mice were purchased from Scanbur AB (Sollentuna, Sweden) and quarantined for one week. Mice were housed in groups of four in stainless steel cages bedded with aspen chip and provided with standard mouse chow diet (Altromin no. 1314 FORTI, Altromin Spezialfutter GmbH & Co., Germany) and tap water ad libitum. The environment of the animal room was carefully controlled, with a 12-h dark/light cycle, temperature of 20-21°C, and relative humidity of 40-45%. ENM stock dispersions were prepared on the same day prior to use in endotoxin free water (HyClone, HyPure Cell Culture Grade Water, Thermo Scientific, Waltham, MA, USA) according to the NANOSOLUTIONS Standard operation protocols (SOPs). Further dilutions were done into phosphate buffered saline (PBS) to mimic physiological conditions. Oropharyngeal aspiration exposures of the ENMs were performed to 8-week old mice under anesthesia using a dose of 10µg/mouse dispersed in 50 µl of PBS once per day on 4 consecutive days. Control mice received plain vehicle. Mice were sacrificed 24 h after the last administration. Immediately after sacrificing, lung tissue samples were collected into RNAlater stabilizing solution (Ambion, Life Technologies, CA, USA) and stored at -80℃. For RNA extraction, the RNAlater-stabilized samples were thawed and homogenized in a Fast prep homogenizer (BIO 101, Thermo Savant, Waltham, MA, USA) using Lysing matrix D, 1.4 mm ceramic spheres (MP Biomedicals, Illkirch, France) and Trisure reagent (Bioline reagents Ltd., London, UK). totRNA was extracted and purified by phenol-chloroform isolation method according to the protocol provided by the manufacturer (Bioline reagents Ltd.). The RNA quality was confirmed by NanoDrop spectrophotometer (ND-1000, Thermo Fischer Scientific Inc., Wilmington, NC, USA) and Bioanalyzer (Agilent technologies, Santa Clara, CA, USA).

#### Bronchoalveolar lavage (BAL) fluid analysis

BAL samples (100 µL) were harvested from mice as previously described^5^ and were cytocentrifuged on slides at 300 g for 10 min (Miles Scientific Cyto-Tek centrifuge, Sakura Finetek). The slides were air-dried and stained with May Grünwald-Giemsa (MGG). BAL cell differentials (number of macrophages, neutrophils, eosinophils and lymphocytes) were obtained as average counts from three high-power fields (HPF). The cells were counted at 50x under light microscopy (Leica DM 4000B; Leica, Wetzlar, Germany). The results will be reported in detail elsewhere (Kinaret *et al*., manuscript in preparation).

#### Microarray assays (*in vitro* and *in vivo* samples)

Samples with RNA integrity values > 7.5 were used for microarray analysis, performed as previously described.^6^ In brief, two total RNA samples were combined into one and 200 ng of the combined sample was labeled with Cy3 or Cy5 (Quick amp labelling kit, two-color, Agilent). Labeled samples were randomly hybridized to Agilent Sure Print G3 Mouse, GE8x60K DNA microarrays, or to Agilent SurePrint G3Human GE 8x60K DNA microarrays, according to the manufacturer’s protocol (Agilent, USA). Hybridized slides were scanned with Agilent microarray scanner (Model G2505C, Agilent, USA) and raw data were extracted using Agilent feature extraction software (V12.0.1.1).

#### microRNA profiling and data analysis

The analysis of microRNAs was performed by applying next-generation sequencing as described previously.^7^ To this end, libraries for smallRNA-seq were prepared from 1 μg of total RNA, containing the smallRNA fraction, with Illumina TruSeq smallRNA sample preparation kit according to the manual. The libraries were size selected to enrich the smallRNAs with size less than 250 bp. The cluster generation was performed automatically with a c-Bot instrument (Illumina). The mRNA-seq libraries were sequenced on the HiSeq2000 platform (Illumina). SmallRNA-seq data were mapped to the [miRBase](https://www.sciencedirect.com/topics/biochemistry-genetics-and-molecular-biology/mirbase) database (build hg19) with Bowtie2 and to the hg19 human reference genome with TopHat2. EdgeR was used to identify the differentially expressed smallRNAs and a minimum expression change of 1.5-fold was used. In addition, an adjusted p value cutoff (0.05) was applied to extract the statistically significant differences.

#### Protein extraction and mass spectrometry

Protein extraction and subsequent proteomics analysis was performed as described. ^8^ Briefly, 1 x 10^6^ cells / well were seeded in a 12-well plate one day before exposure. Cells were treated for 24 h with the determined concentration of ENMs (EC_10_ or a fixed dose for the non-cytotoxic ENMs). Cell culture medium without ENMs was used as negative control, while LPS (100 ng/mL) and STS (EC_50_= 4 µM) were used as positive controls. Cells were harvested and washed twice with PBS at room temperature (directly in the plate for the adherent BEAS-2B cells or by centrifugation in 1.5 mL Eppendorf tubes 300 g, 5 min, room temperature for the non-adherent THP-1 cells) and samples were processed as described previously. ^8^ Pellets were kept at -80°C until analysis by using a nanoflow HPLC combined with Q Exactive Plus Hybrid Quadrupole-Orbitrap mass spectrometer. Data processing was done using Raw2MGF and ClusterMGF from the Quanti workflow[85](https://www.nature.com/articles/s41598-019-40579-6#ref-CR85). The data were searched against the complete human proteome (www.uniprot.org), and quantified by Quanti, a software developed in-house for label-free quantification. Student’s t-test, adjusted for multiple testing using false discovery rate (FDR), was used to compare the different treatments.

#### Protein corona formation and analysis

The so-called “hard” protein corona formed on both bare and surface modified ENMs was prepared by incubating the ENMs at a concentration of 0.1 mg/ml in 80% human plasma solution (total protein content 42 mg/mL) at room temperature for 1 h. After the incubation, samples were spun down and washed three times with 0.5 ml of PBS buffer pH 7.4. The three washing steps allowed for removal of proteins loosely bound to NPs surface and recovered the hard corona composed of the proteins with the greatest affinity. The final pellet was re-suspended in 0.1mL of PBS buffer. The analysis of the hard corona composition was done by Tris-Glycine SDS-PAGE and LC-MS/MS, as described.^9^ The hard corona complexes were separated and denatured by boiling for 5 minutes in blue loading buffer (62.5 mM Tris-HCl (pH 6.8), 2% (w/v) SDS, 10% glycerol, 0.01% (w/v) bromophenol blue, 40 mM DTT). The prepared samples were separated by 10% polyacrylamide gel (1D SDS-PAGE), in electric field using a Mini-PROTEAN Tetra electrophoresis system from Bio-Rad. The electrophoresis was run under constant voltage of 140 V for 45 min. All the gels were stained with silver staining kit. For the LC-MS/MS analysis, the proteins of the corona complexes were first separated by 10% Tris-Glycine SDS-PAGE gel. After running the electrophoresis under constant voltage of 140 V for 10 min, the gel was stained with Commassie blue and proteins bands were taken from each lane prior to trypsin digestion and mass spectrometry. The gel section containing the proteins was removed using a sterile scalpel and transferred to a clean 0.5 mL sample tube which had been pre-rinsed with acetonitrile. The gel sections were trypsin digested in gel. The samples were resuspended in 0.1% w/w formic acid prior to analysis by electrospray liquid chromatography (LC-MS/MS). HPLC-coupled to a Thermofisher Q-Exactive was used to analyze the samples.

#### Omics data processing

In this section we describe the methods employed in the pre-processing of omics data. *mRNA expression profiles*. In brief, mRNA expression raw median values data from agilent microarray data were imported using limma *read.maimages* function. A quantile-based strategy was used to filter out low quality probes based on the value of negative control probes. In particular, probes having a value higher than the 80% quantile of negative control probes in at least two samples were considered for subsequent analytical steps. Expression values were then log(2) transformed and normalized between samples using quantile normalization. No batch effect correction was needed for the THP-1 and BEAS-2B microarray datasets. The ComBat method from package SVA^13^ was used to remove the effect of labeling and slide from the mouse lung microarray dataset. Finally, the log ratio of the average expression levels between each nanomaterial exposure and the corresponding controls was compiled and given in input to the feature selection and classification algorithm. *miRNA expression profiles.* Data processing (miRNA alignment and quantification) was done using the miRDeep2 package^14^. Adapter sequences were removed before the sequence alignment. The data alignment of trimmed reads to a reference genome was then implemented (BWA/miRBase v21). The miRNA expression values were normalized to counts per million (CPM) using trimmed mean of M-values (TMM) normalization from edgeR package^15^. TMM calculates a linear scaling factor, which is the weighted trimmed mean of log expression. *Protein abundance profiles.* The software package MaxQuant was used for analysing shotgun proteomics data. The human complete proteome database (Uniprot) was used for mapping peptides to human proteins (1% FDR, with at least 2 peptides). *Protein corona-based profiles.* Spectra were analysed by label free quantification using MaxQuant 1.4.1.2 software.^16^ A semi-quantitative assessment of the proteins amount was performed by the method of spectral counting (SpC), which represents the total number of the MS/MS spectra for all peptides attributed to a matched protein. The SpC of each protein identity was normalized to the protein mass and expressed as the relative protein quantity.

#### GARBO – parameter setting

GARBO implements a Genetic Algorithm for feature selection and classification. The following text summarizes the set of parameters that was applied for the identified classification tasks.

- Number of generations: 500.
- Number of chromosomes (or individuals): 500.
- Number of niches (or parallel runs): 5.
- Percentage of the best chromosomes exchanged across the niches: 25%.
- Initial number of selected features for each chromosome: [min:30, max 50]
- Saving rate of the population: 20

It should be noted that GARBO, differently from the standard GA approach, is able to dynamically adjust the following GA parameters: crossover probability (starting value = 0.5) and mutation probability (starting value = 0.1).

### SUPPLEMENTARY DISCUSSION

#### Homogenization of different toxicity assay data

Different toxicity end points were assessed to estimate the overall toxicity effects caused by nanomaterials exposures (after 24h) in different *in vitro* models. Cytotoxic effects of the 31 ENMs were measured on BEAS-2B, THP-1, Jurkat (3.5h), and HMDM cells after 24h of exposure, except for Jurkat cells for which the exposure time was set to 3.5h. Then, genotoxic effects, including DNA and chromosomal damages, were measured on BEAS2B cells. A point-based categorization strategy was implemented in order to homogenize the toxicity assay data related to cytotoxicity and genotoxicity data. The supplementary tables 2-4 report a tabular description of the points to be assigned for each toxicity characterization (e.g. reaches 30% toxicity, reaches 50% toxicity, etc.). The term “efficiency” indicates the lowest dose giving a statistically significant increase. The term “efficacy”, on the other hand, indicates the maximum fold-effect in comparison with control. Please, note that the category #1 indicates a Negative response (none significant dose, nor linear-dose response) ; the category #2 indicates an equivocal response (one intermediate significant dose, no linear-dose response; the category #3 indicates an equivocal and positive linear-dose response; and the categories #4-6 indicates a positive response. The supplementary tables 6-8 show an example of homogenization results based on three toxicity end-points.

#### Number of classes definition

The quality of different groupings of the bioactivity variables was evaluated according to the gaussian mixture model with spherical variance and the Bayesian Information Content. For each bioactivity dataset, each different number of clusters is associated with a distribution of BIC, derived from 250 random initializations of the algorithm. Although lower values of the BIC would imply better goodness of fit for a higher number of clusters (i.e. 5, 6), the higher variability of the distribution of the scores for all datasets implies a higher susceptibility to the particular random initialization of the clustering procedure, making less stable any downstream result. For this reason, we decided to only keep the lower numbers of clusters (i.e. 2, 3, 4) which are less driven by the random initialization and are also more suitable to the amount of data samples available.

#### Comparison of Garbo with PCA and LASSO method

The models obtained by means of the Garbo algorithm were compared with those obtained with two alternative multivariate approaches: Logistic Regression with PCA (LR-PCA) and LASSO regularization regression.

PCA: The contribution of each feature to the first two principal components is sensible to dataset perturbation and the classification accuracies are in the range of 35% and 57% for CYT, 20% (BEAS-2B) and 70% (THP-1 proteomics; in vivo CG 60%) for NEU and 30% and 50% for INT.

LASSO: The lasso-based approach, selects a small number of features depending on the dataset and on the amount of regularization. For each single dataset the biomarkers are sensitive to dataset perturbations and they have poor discrimination capability [range 30%-40%]. The instability in the features selected by the LASSO models might indicate correlations between the features.

# Testing biomarkers sensitivity to different toxicity-driven ENM groupings

We assessed the sensitivity of the best performing classification models with respect to different choices in toxicity-driven ENM groupings. The groupings of ENMs, as mentioned above, were obtained by applying unsupervised learning methods on toxicity assay data, and then the optimal number of clusters was selected by using the BIC criterion (Suppl. Fig. S1). Although the optimal number of clusters is three, our analysis suggests that ENM groupings leading to two or four classes would also leverage high prediction performances. Therefore, we tested the RF-based classifiers, which are trained for the classification of ENMs in three groups, on grouping obtained by selecting a different number of optimal clusters (see Suppl. Figure S7). We observed that the selected biomarkers achieved very good classification performances when defining two groups of ENMs (NoL toxicity vs. High toxicity). However, the accuracy decreases when using groupings based on four classes, which might be a result of the limited number of ENMs present in our panel.

# Integrated models for ENMs hazard prediction

An objective of the present study was to verify whether the integration of different omics data types and physical-chemical properties could improve the classification performance of the ENM safety classifier. A model-based integration strategy was applied in order to build a classifier on each individual marker set before combining their model predictions. This strategy offers several benefits: (i) it tolerates missing data layers, and (ii) it allows the integration of data sets in which each data type is available on a different set of samples. The Supplementary Figure 10 summarizes the classification results obtained when combining two, three or four models selected from different data layers.

The selected combinations of data sources aim to highlight differences between classifiers integrating only omics data and classifiers adding physical-chemical properties or corona features. These results provide interesting insight concerning the best exposure setup in the different classification tasks.

When considering the cytotoxicity classification tasks (see Suppl. Figure 3a), all the classifiers derived from single or multiple data layers in THP-1 outperformed those derived from other biological models. Moreover, classification model combining omics-based features achieved the highest prediction scores. In the integrated toxicity task (see Suppl. Figure 3b), classifiers derived from multiple data layers in BEAS-2B outperformed those derived from THP-1 and the *in vivo* derived models. Not surprisingly, mRNA-based biomarker models obtained from mouse lung are the most accurate among other models based on single data layers (see Suppl. Figure 3c). However, classifiers obtained from combinations of omics data layers in BEAS-2B achieved a high classification accuracy when predicting mouse lung neutrophil counts. It is also noted that QSAR-like predictive models, based on intrinsic properties of ENMs alone, never reached a satisfactory range of accuracy (lower than 70%

### SUPPLEMENTARY FIGURES


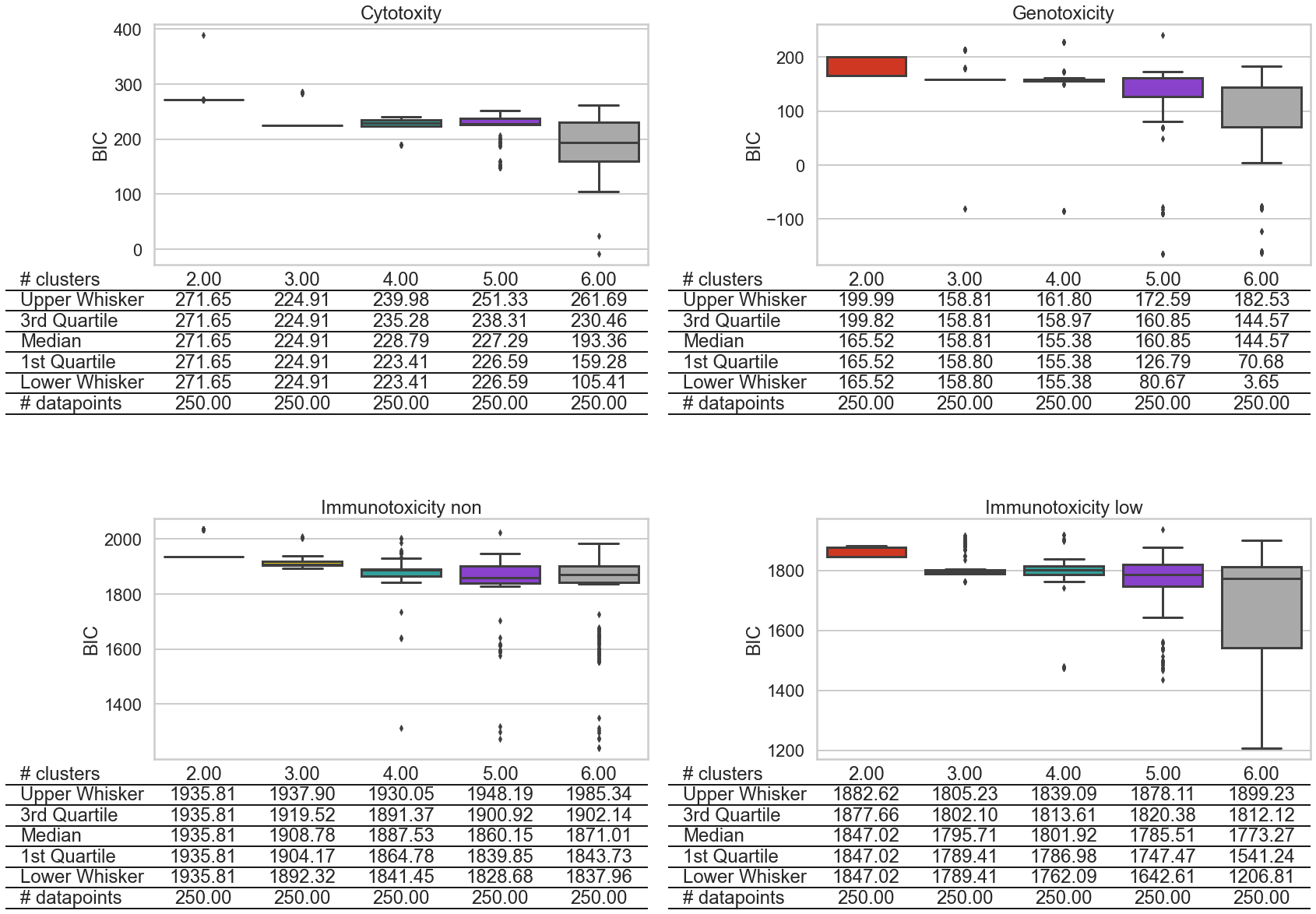


**Supplementary Figure 1 – Identifying the right number of classes.** Barplots showing the distributions of the Bayesian Information Content (BIC) score (n=250) for different numbers of groups into which divide the datasets according to the bioactivity scores.


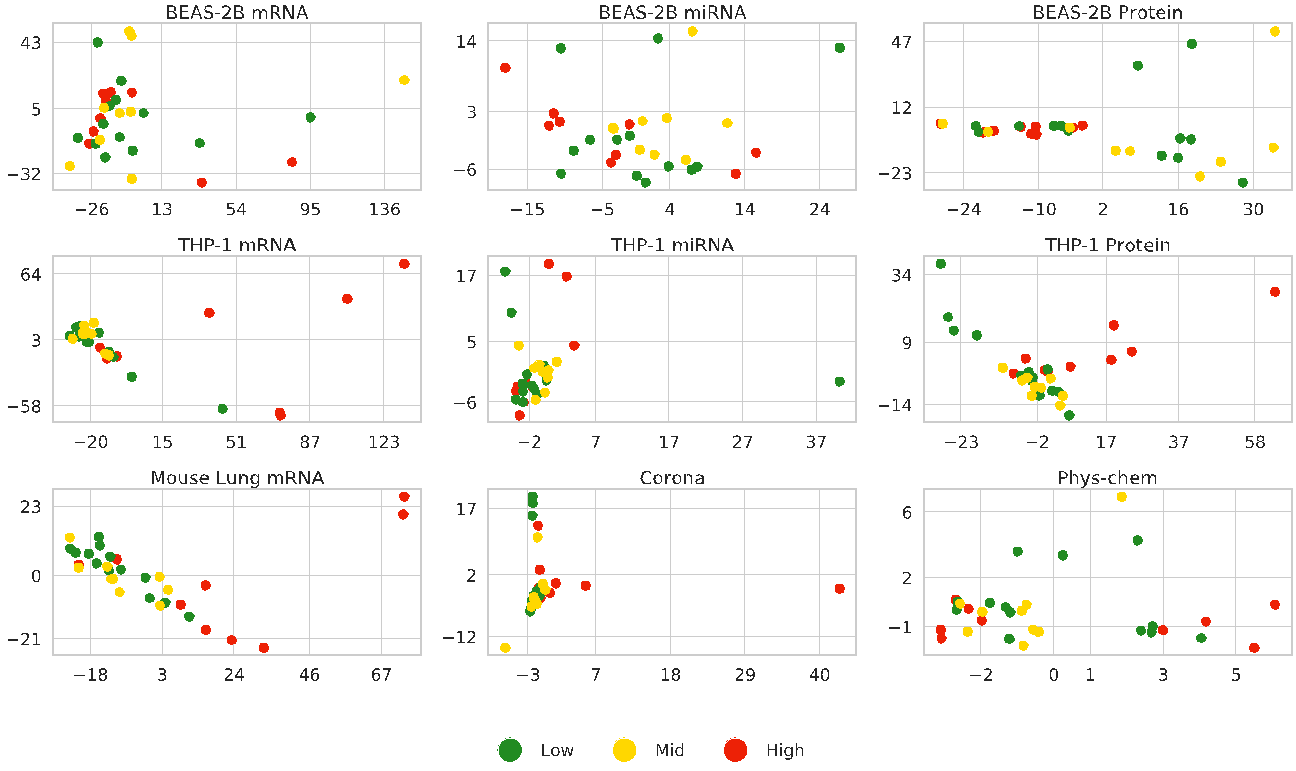


**Supplementary Figure 2 - PCA based exploratory analysis (CYT)**. Scatterplot of the first two principal components of each dataset. Samples are colored according to the grouping generated upon the toxicity endpoints of cytotoxicity. The grouping was generated by using the K-Means algorithm (K=3).


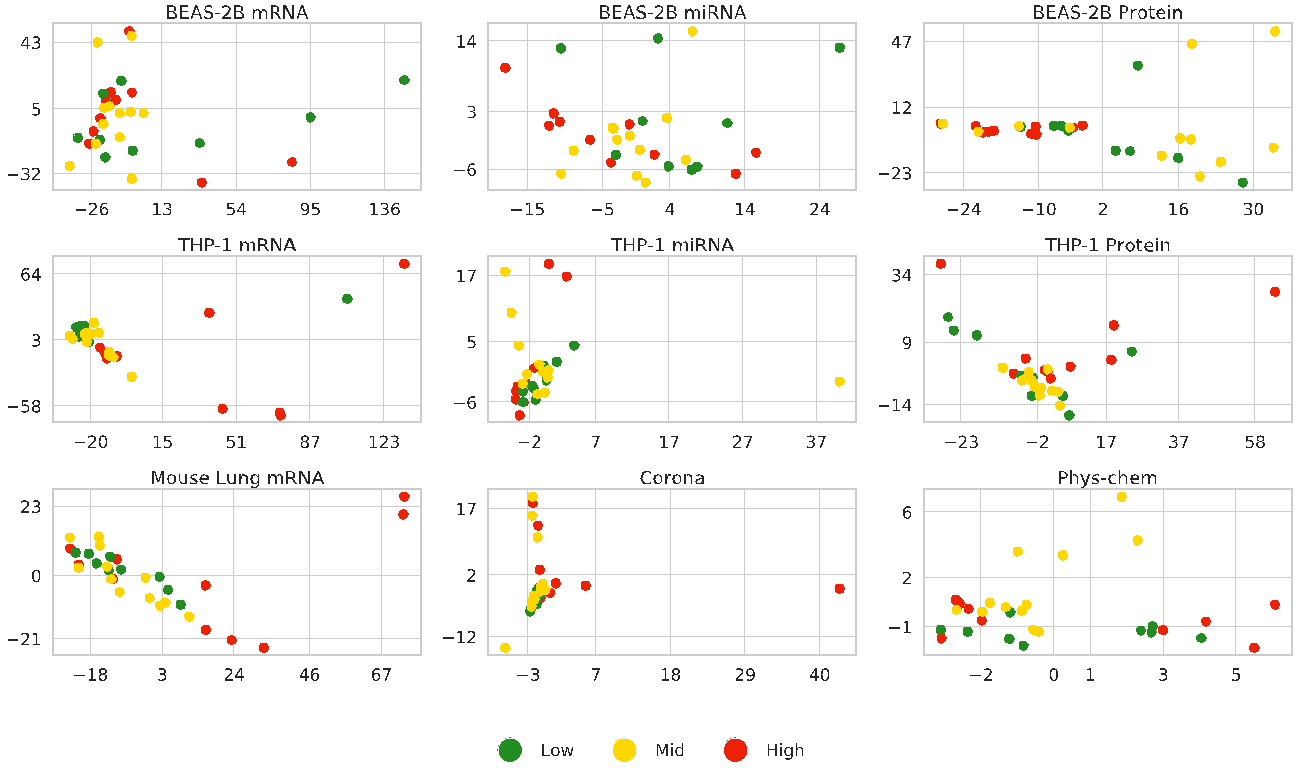


**Supplementary Figure 3 - PCA based exploratory analysis (INT).** Scatterplot of the first two principal components of each dataset. Samples are colored according to the grouping generated upon the integration of toxicity endpoints of cytotoxicity, oxidative stress, immunotoxicity and genotoxicity. The integration was implemented by using a multi-view clustering algorithm (SNF).


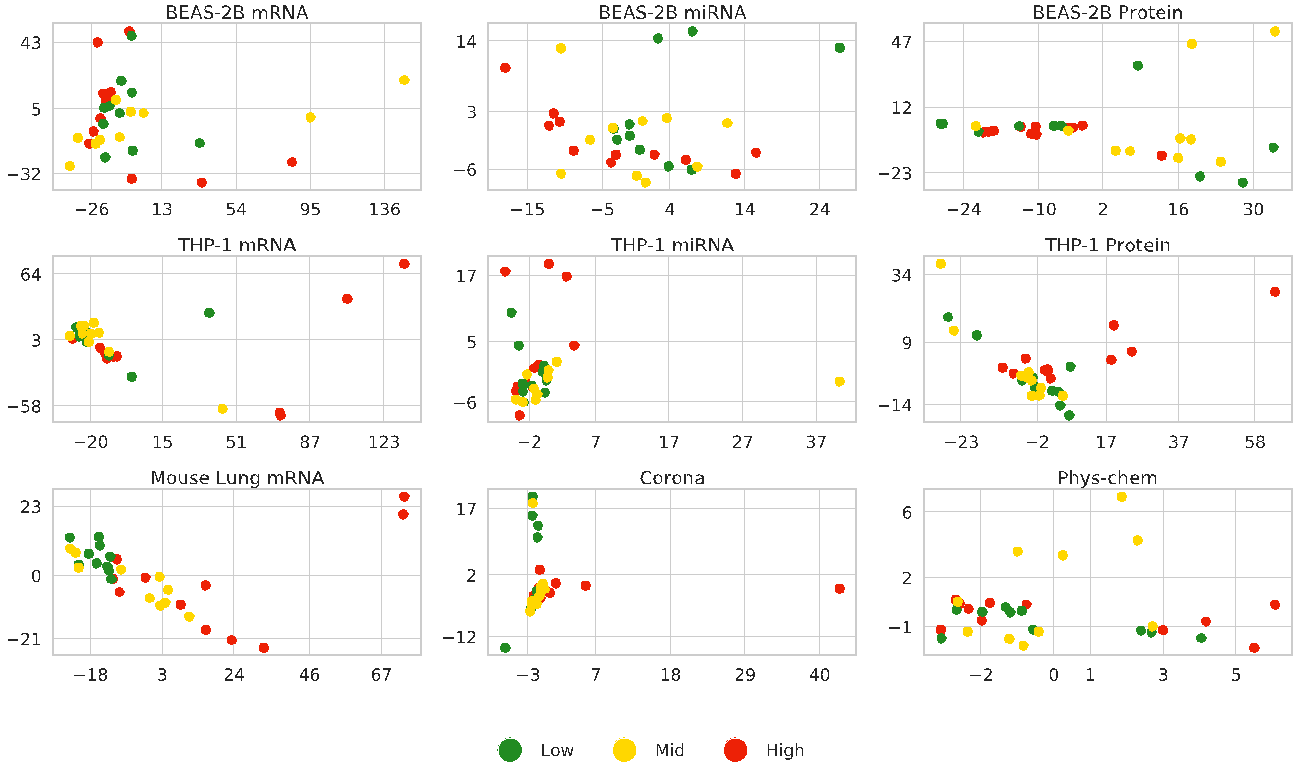


**Supplementary Figure 4 - PCA based exploratory analysis (NEU)**. Scatterplot of the first two principal components of each dataset. Samples are colored according to the grouping generated upon the BAL cell counts that included percentages of neutrophils. The integration was implemented by discretizing the vector of percentages of neutrophils into K bins: equal frequencies (% of the total data, same number of observations per bin)


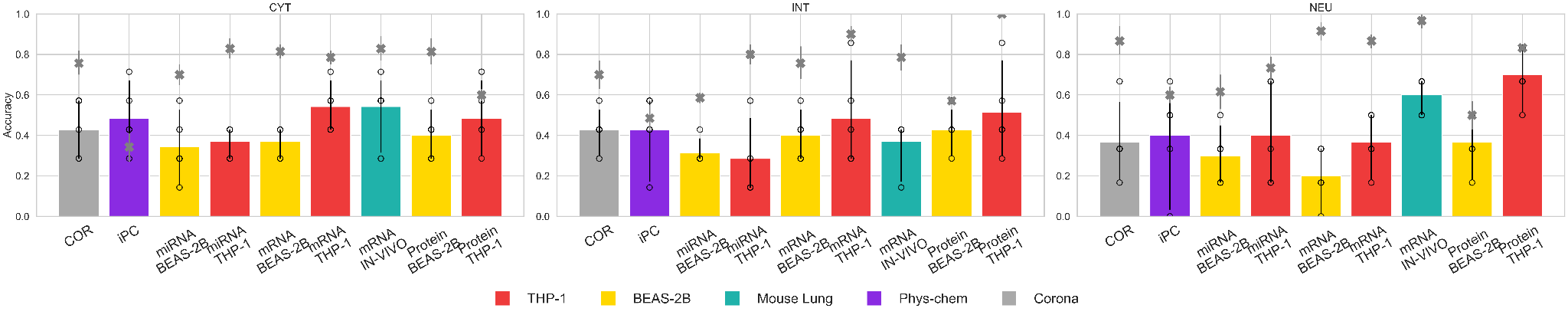


**Supplementary Figure 5 - Classification performances of PCA-LR-based classification models compared to the performances achieved by RF-based classifiers trained on the selected biomarker models.** The symbol x on the top of each bar-plot indicates the accuracy of the best model found by GARBO. N = 5 fold cross validation strategy. Data are represented as mean values and the 95% confidence intervals.


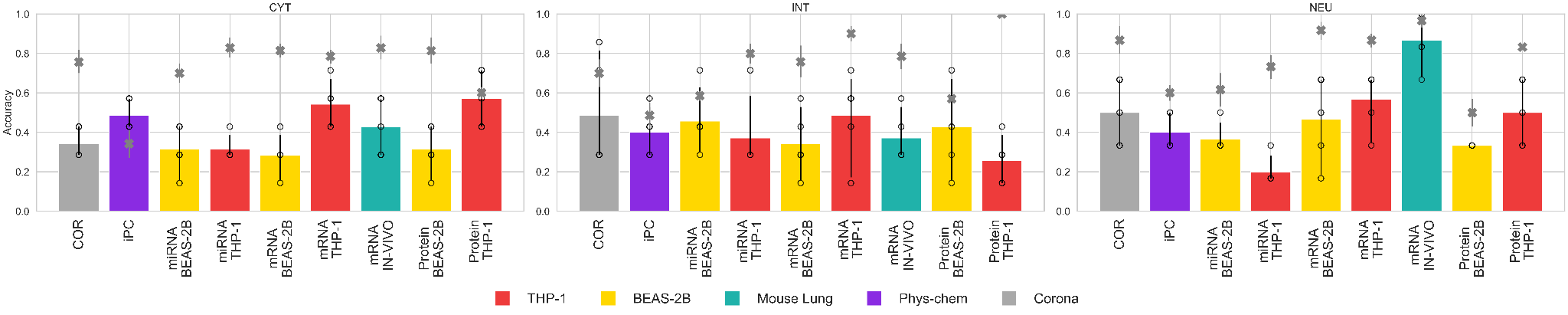


**Supplementary Figure 6 - Classification performances of Lasso-based models compared to the performances achieved by RF-based classifiers trained on the selected biomarker models.** The symbol x on the top of each bar-plot indicates the accuracy of the best model found by GARBO. N = 5 fold cross validation strategy. Data are represented as mean values and the 95% confidence intervals.


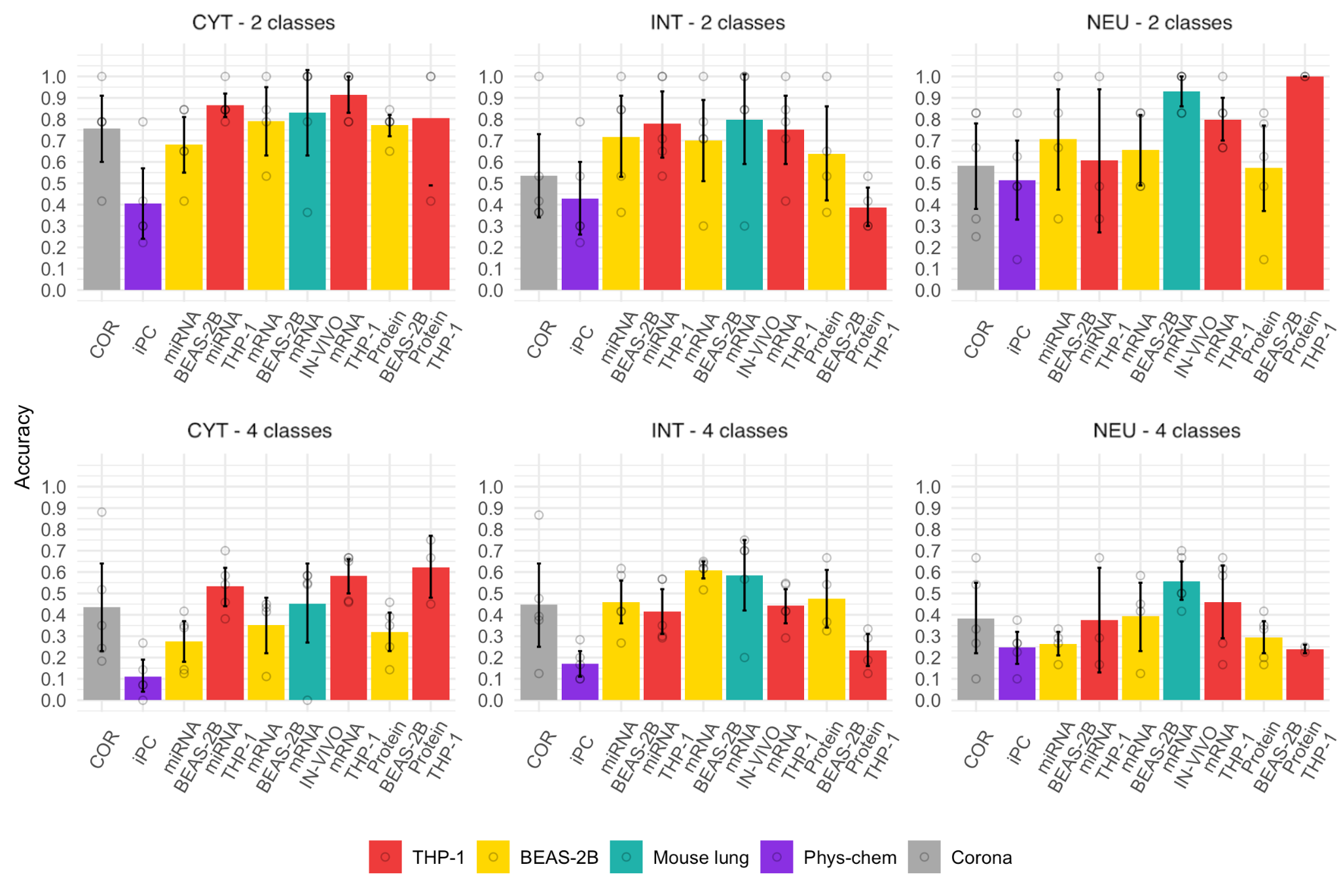


**Supplementary Figure 7 - Classification performances of RF-based trained models that utilize the selected biomarkers to address classification tasks with different levels of granularity.** N = 5 fold cross validation strategy. Data are represented as mean values and the 95% confidence intervals.

**
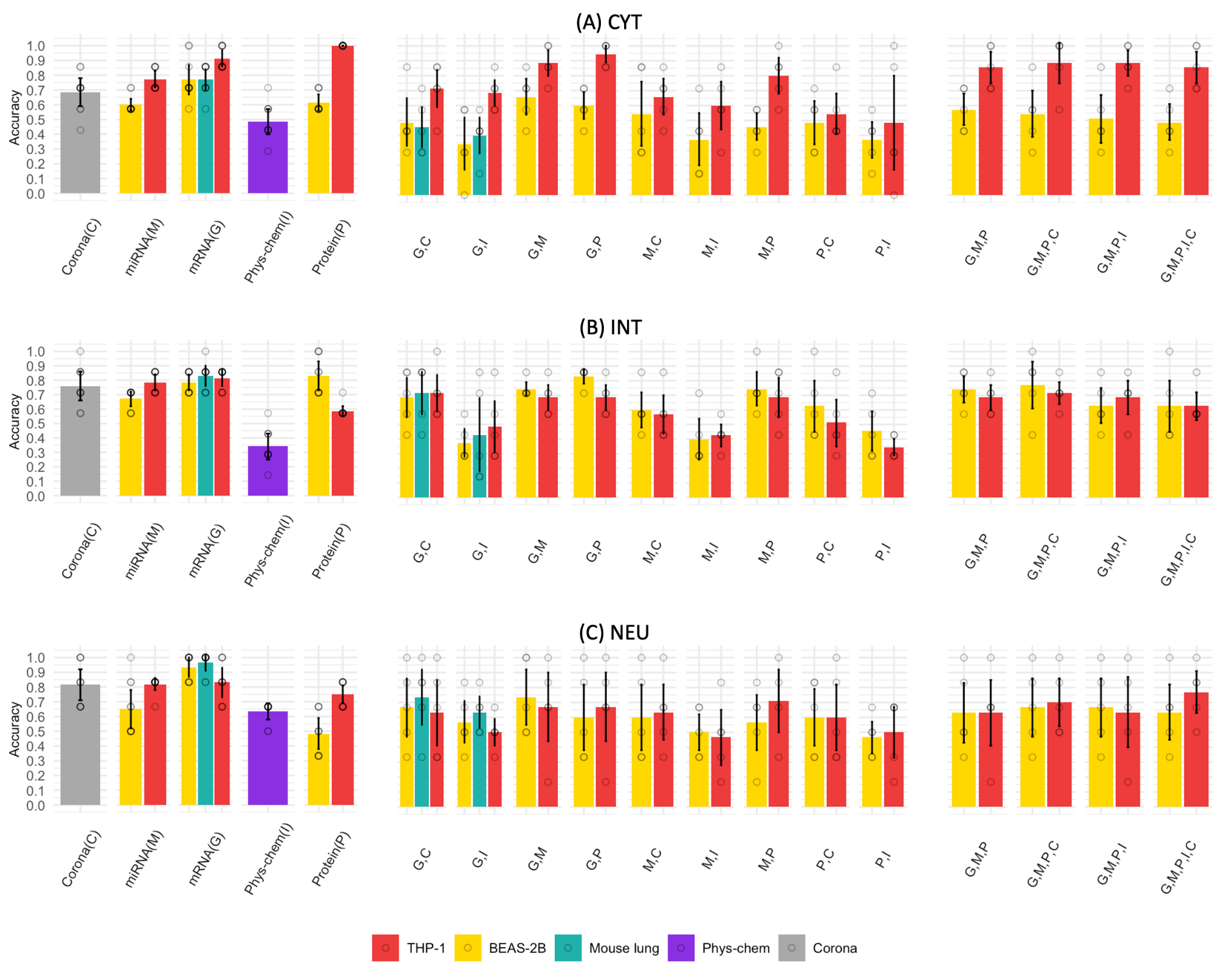
**

**Supplementary Figure 8 - Comparison between single- and multi-view models.** Bar plots comparing the testing accuracy of single omics-based biomarkers (left panel) with that achieved when merging different biomarker models with a late classifier fusion scheme. N = 5 fold cross validation strategy. Data are represented as mean values and the 95% confidence intervals. The late fusion schema combines the output of multiple classifiers, which were trained by using the best biomarker model selected from each data layer. The left panel shows the performances of single omics-based biomarkers. The middle and right panels show the accuracy obtained when combining the best biomarker models from two or three data layers, respectively. (A) Distributions of accuracy values of the models selected for the cytotoxicity score. (B) Distributions of accuracy values of the models selected for the integrated toxicity classification task. (C) Distributions of accuracy values of the models selected for the *in vivo* based toxicity scores. Abbreviations are used to concisely describe the data layers: Corona (or C) - corona data; Physical-chemical properties (or I); mRNA (or G) - gene expression data; miRNA (or M) - miRNA expression data; Protein (P) - proteomics data.


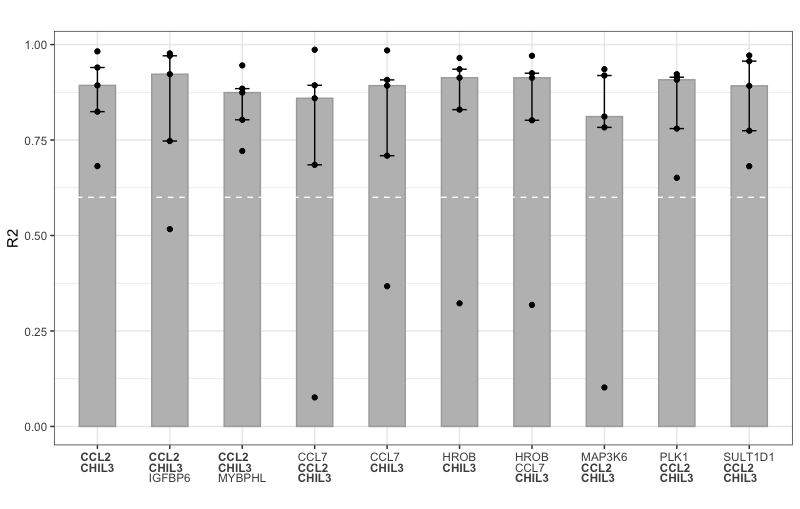


**Supplementary Figure 9 - Feature set capability to predict neutrophils infiltration.** The testing R2 scores, for the top mRNA-based models identified from the mRNA *in vivo* data, for the *in vivo* toxicity based classification task, to predict the neutrophils BAL cell counts. N=5-fold cross validation, error bars represent the 25th and 75th percentiles. Genes in bold are the most frequently selected. The dashed horizontal line indicates an R2 threshold of 0.6.


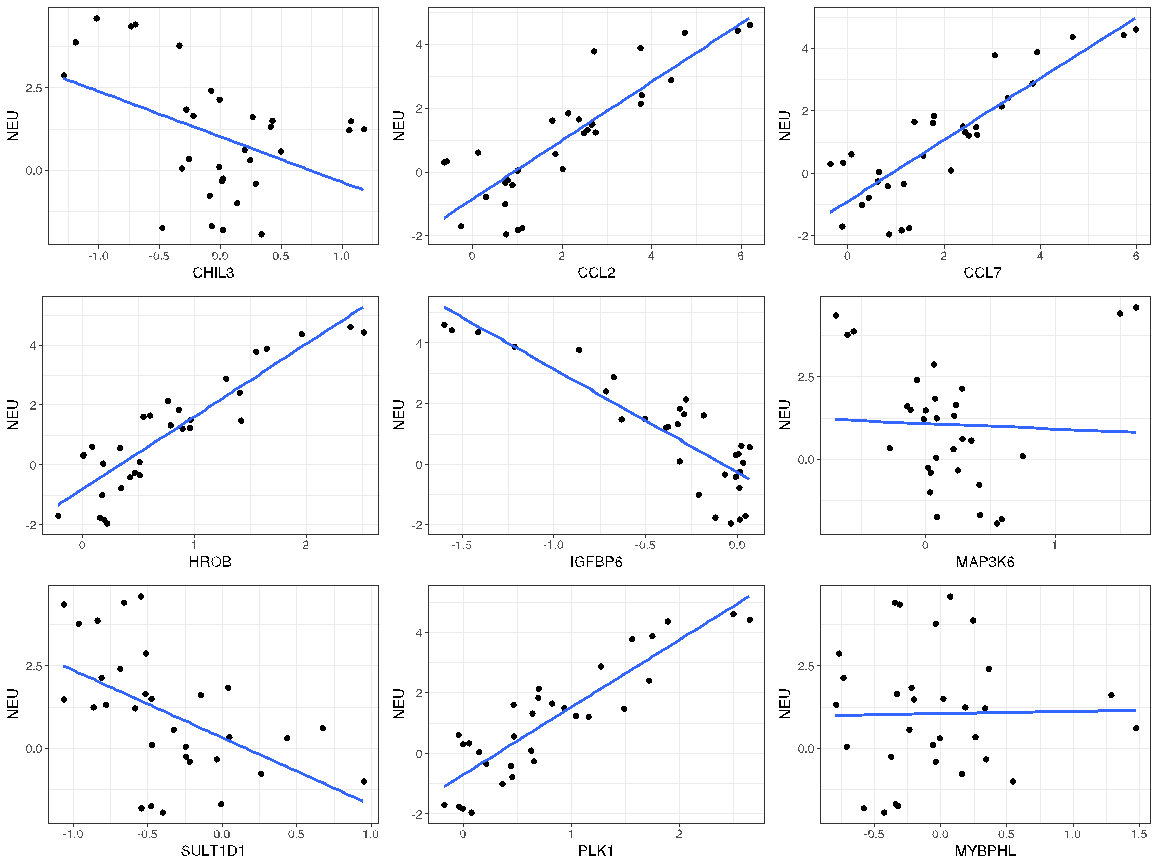


**Supplementary Figure 10 – Scatterplots showing the direction of the associations between two selected genes and the neutrophils BAL cell counts.**


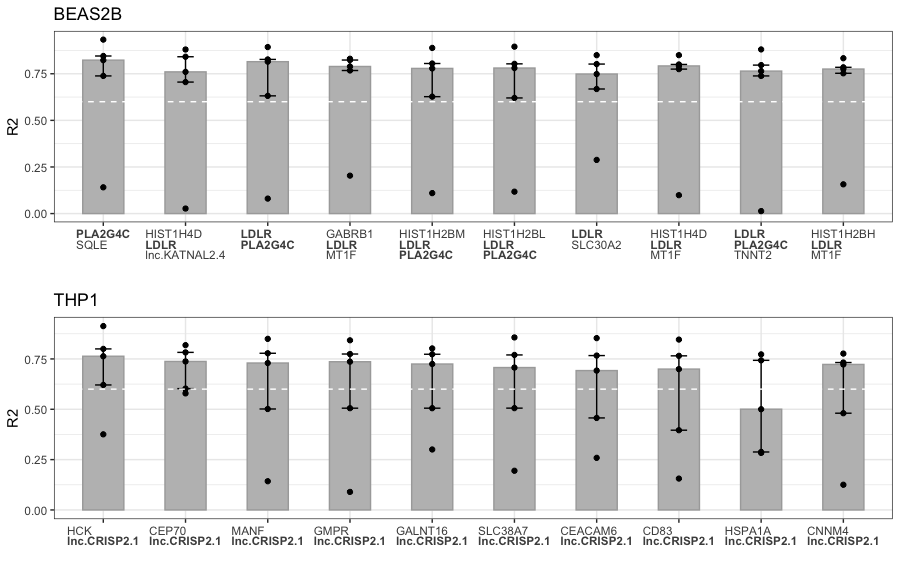


**Supplementary Figure 11:** **capability of in vitro biomarkers to predict neutrophils infiltration.** The testing R2 scores, for the top mRNA-based models from *in vitro* transcriptomics data (generated from BEAS2B (A) and THP1 (B)) , for the *in vivo* toxicity based classification task, to predict the neutrophils BAL cell counts. Genes in bold are the most frequently selected. The dashed horizontal line indicates an R2 threshold of 0.6. N=5-fold cross validation, error bars represent the 25th and 75th percentiles.


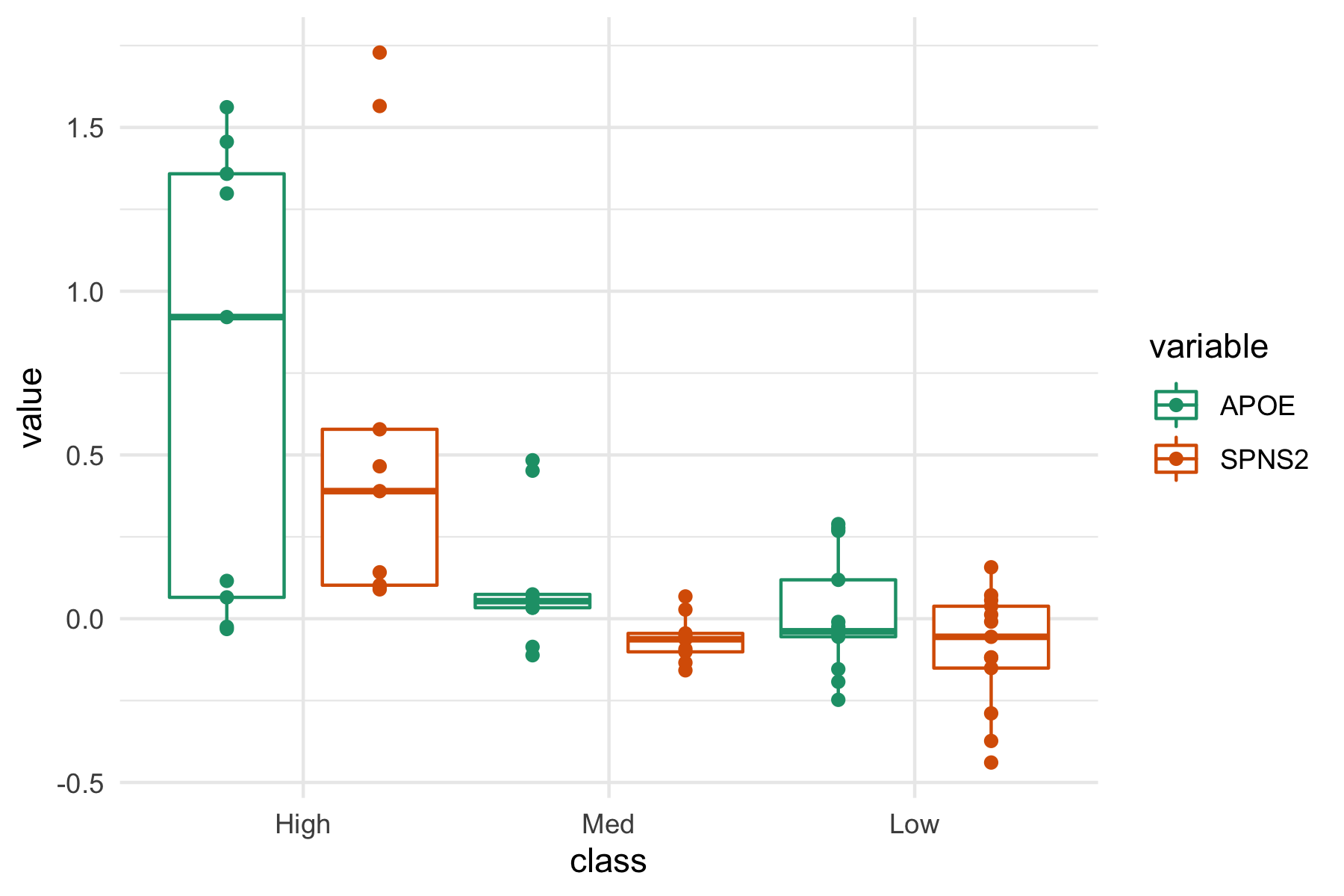


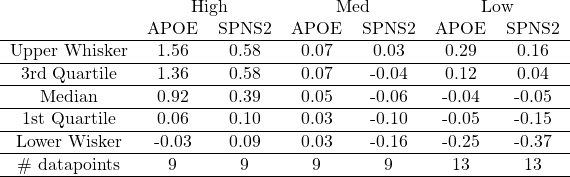


**Supplementary Figure 12 – Boxplots showing the direction of the association between the gene expression profile of APOE and SPNS2 genes and the cytotoxicity classification task based on THP-1 related exposures.** (n = 9 for High, n =9 for Med, n = 13 for Low respectively)


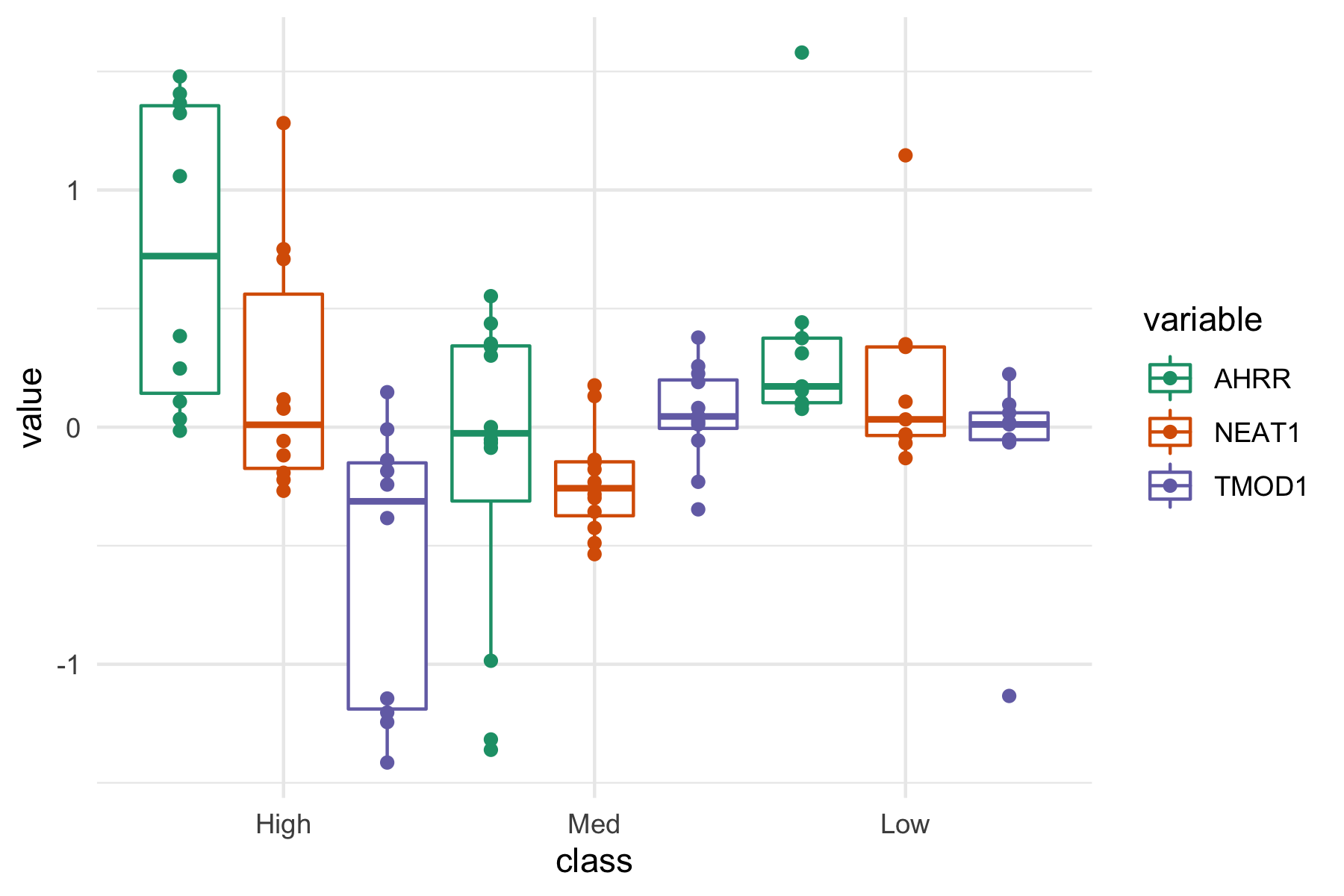


**
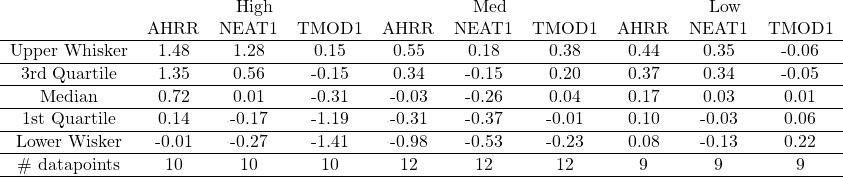
**

**Supplementary Figure 13 – Boxplots showing the direction of the association between the gene expression profile of AHRR, NEAT1 and TMOD1 genes and the integrated classification task based on THP-1 related exposures.** (n = 10 for High, n =12 for Med, n = 9 for Low respectively)

### SUPPLEMENTARY TABLES

**Supplementary Table 1 - List of studied ENMs (included the controls).**

| **Characterised_in_D3.1_20150220** | **NSNP_ID** | **NSNP-Name** | **NSNP_Name_2** | **NSNP_Description** | **NSNP_Base_Substance** | **NSNP_long_descriptive name** | **NSNP_Group** |
| --- | --- | --- | --- | --- | --- | --- | --- |
| Y | NSNP101 | TiO2-Core | TiO2-10to20-Core | TiO2 NP 10 to 20 nm Core | TiO2 | Titanium dioxide nanoparticles 10 to 20 nm Core | TiO2 NPs 10-20 nm |
| Y | NSNP102 | TiO2-Carboxylate | TiO2-10to20-Carboxylate | TiO2 NP 10 to 20 nm Carboxylate -ve functionalised | TiO2 | Titanium dioxide nanoparticles 10 to 20 nm Carboxylate -ve funtionalised | TiO2 NPs 10-20 nm |
| Y | NSNP103 | TiO2-Ammonium | TiO2-10to20-Ammonium | TiO2 NP 10 to 20 nm Ammonium +ve functionalised | TiO2 | Titanium dioxide nanoparticles 10 to 20 nm Ammonium +ve functionalised | TiO2 NPs 10-20 nm |
| Y | NSNP104 | TiO2-PEG | TiO2-10to20-PEG | TiO2 NP 10 to 20 nm PEG | TiO2 | Titanium dioxide nanoparticles 10 to 20 nm PEGylated | TiO2 NPs 10-20 nm |
| Y | NSNP105 | TiO2-rods | TiO2-rods-1x5-ratio | TiO2 rods 1x5 ratio | TiO2 | Titanium dioxide rods 1x5 ratio | TiO2 rods 1x5 ratio |
| Y | NSNP106 | TiO2-rods-Carboxylate | TiO2-rods-1x5-ratio-Carboxylate | TiO2 rods 1x5 ratio Carboxylate -ve functionalised | TiO2 | Titanium dioxide rods 1x5 ratio Carboxylate -ve funtionalised | TiO2 rods 1x5 ratio |
| Y | NSNP107 | TiO2-rods-Ammonium | TiO2-rods-1x5-ratio-Ammonium | TiO2 rods 1x5 ratio Ammonium +ve functionalised | TiO2 | Titanium dioxide rods 1x5 ratio Ammonium | TiO2 rods 1x5 ratio |
| Y | NSNP108 | TiO2-rods-PEG | TiO2-rods-1x5-ratio-PEG | TiO2 rods 1x5 ratio PEG | TiO2 | Titanium dioxide rods 1x5 ratio PEGylated | TiO2 rods 1x5 ratio |
| N | NSNP109 | TiO2-released-sportswear | TiO2-released-sportswear | TiO2 released from sportswear | TiO2 | Titanium dioxide released from sportswear | TiO2 NP Release |
| Y | NSNP201 | Au-5-Carboxylate (COOH) | Au-3to5-Carboxylate | Au NP 3 to 5 nm Carboxylate -ve functionalised | Au | Gold nanoparticles 3 to 5 nm Carboxylate -ve funtionalised | Au particles 3-5 nm |
| Y | NSNP202 | Au-5-Ammonium (NH) | Au-3to5-Ammonium | Au NP 3 to 5 nm Ammonium +ve functionalised | Au | Gold nanoparticles 3 to 5 nm Ammonium +ve functionalised | Au particles 3-5 nm |
| Y | NSNP203 | Au-5-PEG | Au-3to5-PEG | Au NP 3 to 5 nm PEG | Au | Gold nanoparticles 3 to 5 nm PEGylated | Au particles 3-5 nm |
| Y | NSNP204 | Au-20-Carboxylate | Au-10to20-Carboxylate | Au NP 10 to 20 nm Carboxylate -ve functionalised | Au | Gold nanoparticles 10 to 20 nm Carboxylate -ve funtionalised | Au particles 10-20 nm |
| Y | NSNP205 | Au-20-Ammonium | Au-10to20-Ammonium | Au NP 10 to 20 nm Ammonium +ve functionalised | Au | Gold nanoparticles 10 to 20 nm Ammonium +ve functionalised | Au particles 10-20 nm |
| Y | NSNP206 | Au-20-PEG | Au-10to20-PEG | Au NP 10 to 20 nm PEG | Au | Gold nanoparticles 10 to 20 nm PEGylated | Au particles 10-20 nm |
| N | NSNP207 | Au-use-cancer-therapy | Au-use-cancer-therapy | Au NP as used cancer diagnosis & therapy | Au | Gold nanoparticles as used in cancer diagnosis & therapy | Au NP in medical use |
| Y | NSNP301 | CuO-Core | CuO-10to20-Core | CuO NP 10 to 20 nm Core | CuO | Copper oxide nanoparticles 10 to 20 nm Core | CuO NPs 10-20 nm |
| Y | NSNP302 | CuO-Carboxylate | CuO-10to20-Carboxylate | CuO NP 10 to 20 nm Carboxylate -ve functionalised | CuO | Copper oxide nanoparticles 10 to 20 nm Carboxylate -ve funtionalised | CuO NPs 10-20 nm |
| Y | NSNP303 | CuO-Ammonium | CuO-10to20-Ammonium | CuO NP 10 to 20 nm Ammonium +ve functionalised | CuO | Copper oxide nanoparticles 10 to 20 nm Ammonium +ve functionalised | CuO NPs 10-20 nm |
| Y | NSNP304 | CuO-PEG | CuO-10to20-PEG | CuO NP 10 to 20 nm PEG | CuO | Copper oxide nanoparticles 10 to 20 nm PEGylated | CuO NPs 10-20 nm |
| N | NSNP305 | CuO-released-sportswear | CuO-released-sportswear | CuO NP released from sportswear | CuO | Copper oxide nanoparticles released from sportswear | CuO NP Release |
| Y | NSNP401 | Ag-Carboxylate | Ag-10to20-Carboxylate | Ag NP 10 to 20 nm Carboxylate -ve functionalised | Ag | Silver nanoparticles 10 to 20 nm Carboxylate -ve funtionalised | Ag particles 10-20 nm |
| Y | NSNP402 | Ag-Ammonium | Ag-10to20-Ammonium | Ag NP 10 to 20 nm Ammonium +ve functionalised | Ag | Silver nanoparticles 10 to 20 nm Ammonium +ve functionalised | Ag particles 10-20 nm |
| Y | NSNP403 | Ag-PEG | Ag-10to20-PEG | Ag NP 10 to 20 nm PEG | Ag | Silver nanoparticles 10 to 20 nm PEGylated | Ag particles 10-20 nm |
| N | NSNP404 | Ag-released-sportswear | Ag-released-sportswear | Ag released from sportswear | Ag | Silver nanoparticles released from sportswear | Ag NP Release |
| Y | NSNP501 | CdTe-Carboxylate | CdTe-3to5-Carboxylate | CdTe NP 3 to 5 nm Carboxylate -ve functionalised | CdTe | Cadmium telluride NPs 3 to 5 nm Carboxylate -ve functionalised | CdTe particles 3-5 nm |
| Y | NSNP502 | CdTe-Ammonium | CdTe-3to5-Ammonium | CdTe NP 3 to 5 nm Ammonium +ve functionalised | CdTe | Cadmium telluride NPs 3 to 5 nm Ammonium +ve functionalised | CdTe particles 3-5 nm |
| Y | NSNP503 | CdTe-PEG | CdTe-3to5-PEG | CdTe NP 3 to 5 nm PEG | CdTe | Cadmium telluride NPs 3 to 5 nm PEGylated | CdTe particles 3-5 nm |
| N | NSNP504 | CdTe-released-print-ink | CdTe-released-print-ink | CdTe released from printing ink | CdTe | Cadmium telluride released from printing ink | CdTe Release |
| Y | NSNP601 | NanoDiam-Carboxylate | NanoDiam-3to5-Carboxylate | Nanodiamonds 3 to 5 nm Carboxylate -ve functionalised | NanoDiam | Nanodiamonds 3 to 5 nm Carboxylate -ve functionalised | Nanodiamonds 3-5 nm |
| Y | NSNP602 | NanoDiam-Ammonium | NanoDiam-3to5-Ammonium | Nanodiamonds 3 to 5 nm Ammonium +ve functionalised | NanoDiam | Nanodiamonds 3 to 5 nm Ammonium +ve functionalised | Nanodiamonds 3-5 nm |
| Y | NSNP603 | NanoDiam-PEG | NanoDiam-3to5-PEG | Nanodiamonds 3 to 5 nm PEG | NanoDiam | Nanodiamonds 3 to 5 nm PEGylated | Nanodiamonds 3-5 nm |
| N | NSNP604 | NanoDiam-released-oil-additive | NanoDiam-released-oil-additive | Nanodiamonds released from engine oil additive | NanoDiam | Nanodiamonds released from engine oil additive | Nanodiamonds Release |
| Y | NSNP701 | MWCNT-Core | MWCNT-1:100-ratio-Core | MWCNTs 1:100 ratio Core | MWCNT | MWCNTs 1:100 ratio Core | MWCNTs 1x100 ratio |
| Y | NSNP702 | MWCNT-Carboxylate | MWCNT-1:100-ratio-Carboxylate | MWCNTs 1:100 ratio Carboxylate -ve functionalised | MWCNT | MWCNTs 1:100 ratio Carboxylate -ve functionalised | MWCNTs 1x100 ratio |
| Y | NSNP703 | MWCNT-Ammonium | MWCNT-1:100-ratio-Ammonium | MWCNTs 1:100 ratio Ammonium +ve functionalised | MWCNT | MWCNTs 1:100 ratio Ammonium +ve functionalised | MWCNTs 1x100 ratio |
| Y | NSNP704 | MWCNT-PEG | MWCNT-1:100-ratio-PEG | MWCNTs 1:100 ratio PEG | MWCNT | MWCNTs 1:100 ratio PEGylated | MWCNTs 1x100 ratio |
| N | NSNP705 | MWCNT-release-textiles-car-pts | MWCNT-release-textiles-car-pts | MWCNTs released from textile incorporated to car parts | MWCNT | MWCNTs released from textile incorporated to car parts | MWCNT Release |

**Supplementary Table 2 - Point-based system for cytotoxicity end-points.**

| **Cytotoxicity Point system** | Points | |
| --- | --- | --- |
|  | Yes | No |
| Reaches 30% Toxicity | 1 | 0 |
| Reaches 50% Toxicity | 1 | 0 |
| Reaches 70% Toxicity | 1 | 0 |
| IC50%=<100 µg/mL | 2 | 0 |
| IC50%=<50 µg/mL | 2 | 0 |
| IC50%=<20 µg/mL | 2 | 0 |

**Supplementary Table 3 - Point-based system for cytotoxicity end-points.**

| **Chromosomal Damage** | Points | | |
| --- | --- | --- | --- |
|  | Yes | No | Equivocal |
| Positive induction | 3 | 0 | 1 |
| Linear dose-response | 1 | 0 | x |
| Efficiency dose=<100 µg/mL | 1 | 0 | x |
| Efficiency dose=<20 µg/mL | 1 | 0 | x |
| Efficiency dose=<5 µg/mL | 1 | 0 | x |
| Effectivity>2 | 1 | 0 | x |
| Effectivity>4 | 1 | 0 | x |

**Supplementary Table 4 - Point-based system for cytotoxicity end-points.**

| **DNA damage** | Points | | |
| --- | --- | --- | --- |
|  | Yes | No | Equivocal |
| Positive induction | 3 | 0 | 1 |
| Linear dose-response | 1 | 0 | x |
| Efficiency dose=<100 µg/mL | 1 | 0 | x |
| Efficiency dose=<20 µg/mL | 1 | 0 | x |
| Efficiency dose=<5 µg/mL | 1 | 0 | x |
| Effectivity>2 | 1 | 0 | x |
| Effectivity>4 | 1 | 0 | x |

**Supplementary Table 5 - List of categories to be assigned based on the total point-based score.**

|  | Category |
| --- | --- |
| 0 | 1 |
| 1 | 2 |
| 2-3 | 3 |
| 4-5 | 4 |
| 6-7 | 5 |
| 8-9 | 6 |

**Supplementary Table 6 -Cytotoxicity measured on BEAS-2B cells after 24h of exposure.**

| Nanomaterial | Reaches 30% Toxicity | Reaches 50% Toxicity | Reaches 70% Toxicity | IC50%=<100 µg/mL | IC50%=<50 µg/mL | IC50%=<20 µg/mL | Total points |
| --- | --- | --- | --- | --- | --- | --- | --- |
| CuO_core | 1 | 0 | 0 | 0 | 0 | 0 | 1 |
| CuO_NH | 1 | 1 | 1 | 2 | 2 | 2 | 9 |
| CuO_COOH | 1 | 1 | 1 | 2 | 2 | 2 | 9 |
| CuO_PEG | 1 | 0 | 0 | 0 | 0 | 0 | 1 |
| TiO2s_core | 0 | 0 | 0 | 0 | 0 | 0 | 0 |
| TiO2s_NH | 1 | 1 | 1 | 2 | 0 | 0 | 5 |
| TiO2s_COOH | 0 | 0 | 0 | 0 | 0 | 0 | 0 |
| TiO2s_PEG | 1 | 1 | 1 | 2 | 0 | 0 | 5 |
| TiO2r_core | 0 | 0 | 0 | 0 | 0 | 0 | 0 |
| TiO2r_NH | 1 | 1 | 1 | 2 | 0 | 0 | 5 |
| TiO2r_COOH | 0 | 0 | 0 | 0 | 0 | 0 | 0 |
| TiO2r_PEG | 1 | 0 | 0 | 0 | 0 | 0 | 1 |
| MWCNT_core | 1 | 0 | 0 | 0 | 0 | 0 | 1 |
| MWCNT_NH | 0 | 0 | 0 | 0 | 0 | 0 | 0 |
| MWCNT_COOH | 0 | 0 | 0 | 0 | 0 | 0 | 0 |
| MWCNT_PEG | 0 | 0 | 0 | 0 | 0 | 0 | 0 |
| QD_NH | 1 | 1 | 1 | 2 | 2 | 2 | 9 |
| QD_COOH | 1 | 1 | 1 | 2 | 2 | 0 | 7 |
| QD_PEG | 1 | 1 | 1 | 2 | 2 | 0 | 7 |
| Au5_NH | 1 | 1 | 1 | 2 | 2 | 2 | 9 |
| Au5_COOH | 1 | 0 | 0 | 0 | 0 | 0 | 1 |
| Au5_PEG | 0 | 0 | 0 | 0 | 0 | 0 | 0 |
| Au20_NH | 1 | 1 | 1 | 2 | 2 | 2 | 9 |
| Au20_COOH | 1 | 0 | 0 | 0 | 0 | 0 | 1 |
| Au20_PEG | 0 | 0 | 0 | 0 | 0 | 0 | 0 |
| Ag_NH | 1 | 1 | 1 | 2 | 2 | 2 | 9 |
| Ag_COOH | 1 | 0 | 0 | 0 | 0 | 0 | 1 |
| Ag_PEG | 1 | 1 | 1 | 0 | 0 | 0 | 3 |
| ND_NH | 0 | 0 | 0 | 0 | 0 | 0 | 0 |
| ND_COOH | 1 | 0 | 0 | 0 | 0 | 0 | 1 |
| ND_PEG | 1 | 0 | 0 | 0 | 0 | 0 | 1 |

**Supplementary Table 7 -DNA damage measured on BEAS-2B cells after 24h of exposure.**

| Nanomaterial | Positive induction | Linear dose-response | Efficiency dose=<100 µg/mL | Efficiency dose=<20 µg/mL | Efficiency dose=<5 µg/mL | Effectivity>2 | Effectivity>4 | Total points |
| --- | --- | --- | --- | --- | --- | --- | --- | --- |
| CuO_core | 0 | 0 | 0 | 0 | 0 | 0 | 0 | 0 |
| CuO_NH | 0 | 0 | 0 | 0 | 0 | 0 | 0 | 0 |
| CuO_COOH | 0 | 0 | 0 | 0 | 0 | 0 | 0 | 0 |
| CuO_PEG | 0 | 0 | 0 | 0 | 0 | 0 | 0 | 0 |
| TiO2s_core | 1 | 1 | 1 | 0 | 0 | 0 | 0 | 3 |
| TiO2s_NH | 3 | 1 | 1 | 0 | 0 | 1 | 0 | 6 |
| TiO2s_COOH | 0 | 1 | 0 | 0 | 0 | 0 | 0 | 1 |
| TiO2s_PEG | 0 | 0 | 0 | 0 | 0 | 0 | 0 | 0 |
| TiO2r_core | 3 | 1 | 1 | 0 | 0 | 1 | 0 | 6 |
| TiO2r_NH | 0 | 0 | 0 | 0 | 0 | 0 | 0 | 0 |
| TiO2r_COOH | 0 | 0 | 0 | 0 | 0 | 0 | 0 | 0 |
| TiO2r_PEG | 0 | 0 | 0 | 0 | 0 | 0 | 0 | 0 |
| MWCNT_core | 0 | 0 | 0 | 0 | 0 | 0 | 0 | 0 |
| MWCNT_NH | 1 | 1 | 1 | 0 | 0 | 1 | 0 | 4 |
| MWCNT_COOH | 0 | 0 | 0 | 0 | 0 | 0 | 0 | 0 |
| MWCNT_PEG | 0 | 0 | 0 | 0 | 0 | 0 | 0 | 0 |
| QD_NH | 3 | 1 | 1 | 1 | 0 | 1 | 1 | 8 |
| QD_COOH | 0 | 0 | 0 | 0 | 0 | 0 | 0 | 0 |
| QD_PEG | 0 | 1 | 0 | 0 | 0 | 0 | 0 | 1 |
| Au5_NH | 3 | 1 | 1 | 1 | 0 | 1 | 1 | 8 |
| Au5_COOH | 0 | 0 | 0 | 0 | 0 | 0 | 0 | 0 |
| Au5_PEG | 3 | 1 | 0 | 0 | 0 | 1 | 0 | 5 |
| Au20_NH | 3 | 1 | 1 | 1 | 0 | 1 | 1 | 8 |
| Au20_COOH | 0 | 0 | 0 | 0 | 0 | 0 | 0 | 0 |
| Au20_PEG | 0 | 1 | 0 | 0 | 0 | 0 | 0 | 1 |
| Ag_NH | 0 | 1 | 0 | 0 | 0 | 0 | 0 | 1 |
| Ag_COOH | 0 | 0 | 0 | 0 | 0 | 0 | 0 | 0 |
| Ag_PEG | 3 | 1 | 1 | 0 | 0 | 1 | 1 | 7 |
| ND_NH | 0 | 1 | 0 | 0 | 0 | 0 | 0 | 1 |
| ND_COOH | 0 | 1 | 0 | 0 | 0 | 0 | 0 | 1 |
| ND_PEG | 0 | 0 | 0 | 0 | 0 | 0 | 0 | 0 |

**Supplementary Table 8 - Chromosomal damage measured on BEAS-2B cells after 24h of exposure.**

| Nanomaterial | Positive induction | Linear dose-response | Efficiency dose=<100 µg/mL | Efficiency dose=<20 µg/mL | Efficiency dose=<5 µg/mL | Effectivity >2 | Effectivity >4 | Total points |
| --- | --- | --- | --- | --- | --- | --- | --- | --- |
| CuO_core | 3 | 0 | 1 | 1 | 1 | 1 | 0 | 7 |
| CuO_NH | 3 | 0 | 1 | 1 | 0 | 0 | 0 | 5 |
| CuO_COOH | 0 | 0 | 0 | 0 | 0 | 0 | 0 | 0 |
| CuO_PEG | 0 | 1 | 0 | 0 | 0 | 0 | 0 | 1 |
| TiO2s_core | 3 | 1 | 0 | 0 | 0 | 0 | 0 | 4 |
| TiO2s_NH | 0 | 0 | 0 | 0 | 0 | 0 | 0 | 0 |
| TiO2s_COOH | 0 | 0 | 0 | 0 | 0 | 0 | 0 | 0 |
| TiO2s_PEG | 3 | 1 | 1 | 0 | 0 | 0 | 0 | 5 |
| TiO2r_core | 0 | 0 | 0 | 0 | 0 | 0 | 0 | 0 |
| TiO2r_NH | 0 | 0 | 0 | 0 | 0 | 0 | 0 | 0 |
| TiO2r_COOH | 0 | 0 | 0 | 0 | 0 | 0 | 0 | 0 |
| TiO2r_PEG | 0 | 0 | 0 | 0 | 0 | 0 | 0 | 0 |
| MWCNT_core | 0 | 0 | 0 | 0 | 0 | 0 | 0 | 0 |
| MWCNT_NH | 0 | 0 | 0 | 0 | 0 | 0 | 0 | 0 |
| MWCNT_COOH | 1 | 1 | 1 | 0 | 0 | 0 | 0 | 3 |
| MWCNT_PEG | 0 | 0 | 0 | 0 | 0 | 0 | 0 | 0 |
| QD_NH | 3 | 0 | 1 | 1 | 1 | 1 | 1 | 8 |
| QD_COOH | 3 | 1 | 1 | 1 | 1 | 1 | 0 | 8 |
| QD_PEG | 3 | 1 | 1 | 1 | 1 | 1 | 0 | 8 |
| Au5_NH | 3 | 1 | 1 | 0 | 0 | 1 | 0 | 6 |
| Au5_COOH | 0 | 0 | 0 | 0 | 0 | 0 | 0 | 0 |
| Au5_PEG | 0 | 0 | 0 | 0 | 0 | 0 | 0 | 0 |
| Au20_NH | 0 | 0 | 0 | 0 | 0 | 0 | 0 | 0 |
| Au20_COOH | 1 | 0 | 1 | 1 | 1 | 0 | 0 | 4 |
| Au20_PEG | 3 | 1 | 1 | 1 | 1 | 1 | 0 | 8 |
| Ag_NH | 0 | 0 | 0 | 0 | 0 | 0 | 0 | 0 |
| Ag_COOH | 1 | 0 | 1 | 1 | 1 | 0 | 0 | 4 |
| Ag_PEG | 3 | 0 | 1 | 1 | 1 | 1 | 0 | 7 |
| ND_NH | 0 | 0 | 0 | 0 | 0 | 0 | 0 | 0 |
| ND_COOH | 0 | 0 | 0 | 0 | 0 | 0 | 0 | 0 |
| ND_PEG | 0 | 0 | 0 | 0 | 0 | 0 | 0 | 0 |

**Supplementary Table 9 – Description of the external mRNA datasets that were used for the validation of the mRNA-based classification models.**

| **GEO ID** | **Description** | **Nanomaterial** | **Cell model** | **Reference** |
| --- | --- | --- | --- | --- |
| GSE29042 | Mice were aspirated with multiwalled carbon nanotubes in four doses  ranging from 10 µg to 80 µg, plus control, and sacrificed after 1, 7, 28,  and 56 days. RNA was extracted from the lungs and expression profilling  by microarray was performed. | MWCNT | Mm lung | ^17^ |
| GSE35193 | Female mice exposed to 0.018, 0.054 and 0.162 mg Printex 90  carbon black nanoparticles by single intra-tracheal instillation. | Carbon black (Printex® 90) | Mm lung | ^18^ |
| GSE41041 | Pulmonary instillation of low doses of titanium dioxide nanoparticles in mice. This experiment consists of three different dosages of TiO2, e.g., low (18 ug),  medium (54 ug) and high (162 ug), and one control. | TiO2 | Mm lung | ^19^ |
| GSE42067 | Three human cell types respond to multi-walled carbon nanotubes and  titanium dioxide nanobelts with cell-specific transcriptomic and proteomic  expression (SAE) | MWCNT / TiO2-nanobelt | SAE | ^20^ |
| GSE42068 | Three human cell types respond to multi-walled carbon nanotubes and  titanium dioxide nanobelts with cell-specific transcriptomic and proteomic  expression (THP1) | MWCNT / TiO2-nanobelt | THP1 | ^20^ |
| GSE46998 | Transcriptomic analysis of mouse lung tissue exposed to multiwalled carbon  nanotubes (Mitsui7) | MWCNT-Mitsui7 | Mm lung | ^21^ |
| GSE46999 | Transcriptomic analysis of cultured lung epithelial cells exposed to  multiwalled carbon nanotubes (Mitsui7) | MWCNT-Mitsui7 | FE1 | ^21^ |
| GSE50176 | Mice were exposed to rCNT or tCNT for 4 h and sacrificed immediately or  24 h after the exposure. | rCNT/tCNT | Mm lung |  |
| GSE51186 | Transcriptome study of THP-1 human monocytes following exposure for  4 h or 24 h to 50 uM S-Nitrosoglutathione, 50 and 200 ug/ml  S-Nitrosoglutathione-loaded polymeric and empty Eudragit RL nanoparticles | GSNO-polymeric / GSNO | THP1 | ^22^ |
| GSE51417 | To identify the molecular impact of SPIO nanoparticle inhalation exposure  on lung tissue.Transcriptional responses were measured by global microarray  analysis of mouse lung. | SPIO | Mm lung |  |
| GSE53765 | Exposure of human A549 and THP-1 cells and mouse J774 cells to single  walled carbon nanotubes. Cell lines were treated for 24 hours. | SWCNT | A549 | ^23^ |
| GSE55286 | This experiment examined the pulmonary transcriptional response of female   C57BL/6 mice exposed to NRCWE-26, a short multi-walled carbon nanotube,  and NM-401, a long multi-walled carbon nanotube, at three doses:  D1 (18 μg), D2 (54 μg), D3 (162 μg), and vehicle control.  Each dose group was examined 1, 3 or 28 days post-exposure. | MWCNT | Mm lung | ^24^ |
| GSE60797 | Female C57BL/6 mice of 5-7 weeks old were exposed to 18, 54, or 162 ug of  Indoor-NanoTiO2 and Indoor-R dispersed in 10% bronchoalveolar lavage  fluid (BAL), 0.9% NaCl. Each exposure group consisted of minimum of  5 animals. Controls were exposed to vehicle only.  Each dose group was examined 1, 3 or 28 days post-exposure. | TiO2 | Mm lung |  |
| GSE60798 | Adult C57BL/6 mice were exposed via single intratracheal instillation to three   doses of NRCWE-001 (10 nm, neutral charge) NRCWE-002 ( 10 nm, positive charge). Each dose group was examined 1, 3 or 28 days  post-exposure. | TiO2 | Mm lung |  |
| GSE60799 | Adult C57BL/6 mice were exposed via single intratracheal instillation to three  doses of NRCWE-030 (10.5 nm) and NRCWE-025 (38 nm). Each dose group  was examined 1, 3 or 28 days  post-exposure. | TiO2 | Mm lung |  |
| GSE63552 | EAS 2B cells were exposed to 0.25 and 2 μg/cm2 of MWCNT, crocidolite  asbestos and glass wool (MMVF10) for 1, 4, 6, 12, 24 and 48 hours. | Crocidolite/ Glasswool /  MWCNT-Mitsui7 | BEAS-2B | ^25^ |
| GSE81565 | This experiment examined the the lung transcriptomic responses to single  intratracheal instillation of rutile hydrophilic TiO2NPs of 20 nm diameter.  Four different doses were selected:18, 54, 162, or 486 µg/animal.  Samples were collected at day 1, day 28, and day 90 post-exposure | TiO2-rutilehydrophilic | Mm lung | ^26^ |
| GSE81566 | This experiment examined the the lung transcriptomic responses to single  intratracheal instillation of rutile hydrophobic TiO2NPs of 20 nm diameter.  Four different doses were selected:18, 54, 162, or 486 µg/animal. Samples  were collected at day 1, day 28, and day 90 post-exposure | TiO2-rutilehydrophobic | Mm lung | ^26^ |
| GSE81567 | This experiment examined the the lung transcriptomic responses to single  intratracheal instillation of mix rutile/anatase TiO2NPs of 20 nm diameter.  Four diferent doses were selected:18, 54, 162, or 486 µg/animal. Samples  were collected at day 1, day 28, and day 90 post-exposure | TiO2-rutile & anatasemix | Mm lung | ^26^ |
| GSE81568 | This experiment examined the the lung transcriptomic responses to single intratracheal instillation of anatase TiO2NPs of 300 nm diameter.  Four different doses were selected:18, 54, 162, or 486 µg/animal.  Samples were collected at day 1, day 28, and day 90 post-exposure | TiO2-anatase-300nm | Mm lung | ^26^ |
| GSE81569 | This experiment examined the the lung transcriptomic responses to single  intratracheal instillation of anatase TiO2NPs of 8 nm diameter.  Four different doses were selected: 18, 54, 162, or 486 µg/animal.  Samples were collected at day 1, day 28, and day 90 post-exposure | TiO2-anatase-8nm | Mm lung | ^26^ |
| GSE83516 | Primary human monocyte-derived macrophages (HMDM) as a model were  treated with GO (graphene oxide), HCS (hollow carbon spheres) and SWCNT  (single-walled carbon nanotubes) at concentrations from 10-100 µg/ml for 6 or 24 hours. | CarbonSphere/ Graphene Oxide /  SWCNT | HMDM |  |

**Supplementary Table 10 – List of mRNA- and “*in vitro”* based biomarker models for *in vitro* related classification tasks (CYT and INT).**

| Cell Model | Classification Task | Model | Selected features/genes |
| --- | --- | --- | --- |
| THP-1 | INT | 1 | "NEAT1,RET" |
| THP-1 | INT | 2 | "AHRR,MITF,NEAT1,TMOD1" |
| THP-1 | INT | 3 | "AHRR,CCDC172,NEAT1,TMOD1" |
| THP-1 | INT | 4 | "AHRR,NEAT1,TMOD1,lnc.SNX18.1” |
| THP-1 | INT | 5 | "AHRR,IFI44,NEAT1,TMOD1" |
| THP-1 | INT | 6 | "KIF17,NEAT1,TMOD1" |
| THP-1 | INT | 7 | "AHRR,ANXA1,NEAT1,TMOD1" |
| THP-1 | INT | 8 | "AHRR,NEAT1,RAB33A,TMOD1" |
| THP-1 | INT | 9 | "NEAT1,TMOD1" |
| THP-1 | INT | 10 | "AHRR,NEAT1,RET" |
| BEAS-2B | INT | 1 | "CELF6,HCAR3,PTX3,TRIM16L" |
| BEAS-2B | INT | 2 | "PTX3,SPRR1B,TRIM16L" |
| BEAS-2B | INT | 3 | "CELF6,CYR61,HCAR3,KRT6B,TRIM16L" |
| BEAS-2B | INT | 4 | "PIM1,PTX3,TRIM16L" |
| BEAS-2B | INT | 5 | "CELF6,PTX3,TRIM16L" |
| BEAS-2B | INT | 6 | "HCAR3,HSPA6,TRIM16L,lnc.FKBP3.3" |
| BEAS-2B | INT | 7 | "HCAR3,XLOC_l2_015760" |
| BEAS-2B | INT | 8 | "KRTAP3.3,PTX3,TRIM16L" |
| BEAS-2B | INT | 9 | "HCAR3,HSPA6,TRIM16L" |
| BEAS-2B | INT | 10 | "CELF6,HCAR3,TNFAIP6,TRIM16L" |
| THP-1 | CYT | 1 | "APOE,FBXO32,SPNS2” |
| THP-1 | CYT | 2 | "APOE,CTH" |
| THP-1 | CYT | 3 | "APOE,DEPTOR,SPNS2" |
| THP-1 | CYT | 4 | "APOE,SNAI3,lnc.MAP2K6.2" |
| THP-1 | CYT | 5 | "APOE,HYI" |
| THP-1 | CYT | 6 | "APOE,DNAJC6" |
| THP-1 | CYT | 7 | "APOE,CUL1" |
| THP-1 | CYT | 8 | "APOE,TTBK1" |
| THP-1 | CYT | 9 | "APOE,SPNS2" |
| THP-1 | CYT | 10 | "APOE,DEFA4" |
| BEAS-2B | CYT | 1 | "ELF3,RNA18S5,SLC30A2" |
| BEAS-2B | CYT | 2 | "ALDH3A1,SLC6A9" |
| BEAS-2B | CYT | 3 | "MT1F,SLC6A9" |
| BEAS-2B | CYT | 4 | "IFI44,SLC6A9,lnc.KIF25.2" |
| BEAS-2B | CYT | 5 | "MT1E,SLC6A9” |
| BEAS-2B | CYT | 6 | "ELF3,IFI44,SLC30A2" |
| BEAS-2B | CYT | 7 | "MT1X,SLC6A9" |
| BEAS-2B | CYT | 8 | “RNA18S5,SLC30A2” |
| BEAS-2B | CYT | 9 | "IFI44,MT1E" |
| BEAS-2B | CYT | 10 | "IFI44,SLC6A9" |

**Supplementary Table 11 – List of mRNA- and “*in vivo”*-based biomarker models for *in vitro* related classification tasks (CYT and INT) and the classification task NEU.** The models are based on gene expression data from mouse lung tissues.

| Classification Task | Model | Selected features/genes  (Ensemble gene ID, Gene Symbol) |
| --- | --- | --- |
| NEU | 1 | ENSMUSG00000029273 Sult1d1  ENSMUSG00000035385 Ccl2  ENSMUSG00000040809 Chil3 |
| NEU | 2 | ENSMUSG00000030867 Plk1  ENSMUSG00000035385 Ccl2  ENSMUSG00000040809 Chil3 |
| NEU | 3 | ENSMUSG00000035385 Ccl2  ENSMUSG00000040809 Chil3  ENSMUSG00000068745 Mybphl |
| NEU | 4 | ENSMUSG00000034773 Hrob  ENSMUSG00000040809 Chil3 |
| NEU | 5 | ENSMUSG00000034773 Hrob  ENSMUSG00000035373 Ccl7  ENSMUSG00000040809 Chil3 |
| NEU | 6 | ENSMUSG00000035373 Ccl7  ENSMUSG00000040809 Chil3 |
| NEU | 7 | ENSMUSG00000028862 Map3k6  ENSMUSG00000035385 Ccl2  ENSMUSG00000040809 Chil3 |
| NEU | 8 | ENSMUSG00000035385 Ccl2  ENSMUSG00000040809 Chil3 |
| NEU | 9 | ENSMUSG00000035373 Ccl7  ENSMUSG00000035385 Ccl2  ENSMUSG00000040809 Chil3 |
| NEU | 10 | ENSMUSG00000023046 Igfbp6  ENSMUSG00000035385 Ccl2  ENSMUSG00000040809 Chil3 |
| INT | 1 | ENSMUSG00000039519 Cyp7b1  ENSMUSG00000020641 Rsad2 |
| INT | 2 | ENSMUSG00000039519 Cyp7b1  ENSMUSG00000069805 Fbp1 |
| INT | 3 | ENSMUSG00000070529 Wfdc10  ENSMUSG00000020641 Rsad2 |
| INT | 4 | ENSMUSG00000039519 Cyp7b1  ENSMUSG00000053765 Oas1f |
| INT | 5 | ENSMUSG00000039977 Deup1  ENSMUSG00000047222 Rnase2a  ENSMUSG00000020641 Rsad2 |
| INT | 6 | ENSMUSG00000038801 Scgb1c1  ENSMUSG00000020641 Rsad2 |
| INT | 7 | ENSMUSG00000069805 Fbp1  ENSMUSG00000020641 Rsad2 |
| INT | 8 | ENSMUSG00000029053 Prkcz  ENSMUSG00000039519 Cyp7b1 |
| INT | 9 | ENSMUSG00000047222 Rnase2a  ENSMUSG00000070529 Wfdc10  ENSMUSG00000020598 Nrcam |
| INT | 10 | ENSMUSG00000070529 Wfdc10  ENSMUSG00000020089 Ppa1  ENSMUSG00000020641 Rsad2 |
| CYT | 1 | ENSMUSG00000038801 Scgb1c1  ENSMUSG00000020641 Rsad2 |
| CYT | 2 | ENSMUSG00000069805 Fbp1  ENSMUSG00000020641 Rsad2 |
| CYT | 3 | ENSMUSG00000019874 Fabp7  ENSMUSG00000020641 Rsad2 |
| CYT | 4 | ENSMUSG00000025936 Gm4956  ENSMUSG00000020641 Rsad2 |
| CYT | 5 | ENSMUSG00000070529 Wfdc10  ENSMUSG00000074141 Il4i1  ENSMUSG00000074141 Il4i1b  ENSMUSG00000020641 Rsad2 |
| CYT | 6 | ENSMUSG00000043631 Ecm2  ENSMUSG00000070529 Wfdc10  ENSMUSG00000020641 Rsad2 |
| CYT | 7 | ENSMUSG00000066108 Muc5b  ENSMUSG00000020641 Rsad2 |
| CYT | 8 | ENSMUSG00000070529 Wfdc10  ENSMUSG00000020641 Rsad2 |
| CYT | 9 | ENSMUSG00000029379 Cxcl3  ENSMUSG00000020641 Rsad2 |
| CYT | 10 | ENSMUSG00000028633 Ctps  ENSMUSG00000070529 Wfdc10  ENSMUSG00000020641 Rsad2 |

**Supplementary Table 12 – List of mRNA- and “*in vitro”*-based biomarker models for the *in vivo* related classification task (NEU).**

| Cell Model | Classification Task | Model | Selected features/genes |
| --- | --- | --- | --- |
| THP-1 | NEU | 1 | CEACAM6,lnc.CRISP2.1" |
| THP-1 | NEU | 2 | HCK,lnc.CRISP2.1 |
| THP-1 | NEU | 3 | CNNM4,lnc.CRISP2.1 |
| THP-1 | NEU | 4 | CEP70,lnc.CRISP2.1 |
| THP-1 | NEU | 5 | CD83,lnc.CRISP2.1 |
| THP-1 | NEU | 6 | MANF,lnc.CRISP2.1 |
| THP-1 | NEU | 7 | GMPR,lnc.CRISP2.1 |
| THP-1 | NEU | 8 | GALNT16,lnc.CRISP2.1 |
| THP-1 | NEU | 9 | HSPA1A,lnc.CRISP2.1 |
| THP-1 | NEU | 10 | SLC38A7,lnc.CRISP2.1 |
| BEAS-2B | NEU | 1 | LDLR,PLA2G4C,TNNT2 |
| BEAS-2B | NEU | 2 | HIST1H4D,LDLR,lnc.KATNAL2.4 |
| BEAS-2B | NEU | 3 | LDLR,PLA2G4C |
| BEAS-2B | NEU | 4 | PLA2G4C,SQLE |
| BEAS-2B | NEU | 5 | GABRB1,LDLR,MT1F |
| BEAS-2B | NEU | 6 | LDLR,SLC30A2 |
| BEAS-2B | NEU | 7 | HIST1H2BL,LDLR,PLA2G4C |
| BEAS-2B | NEU | 8 | HIST1H2BH,LDLR,MT1F |
| BEAS-2B | NEU | 9 | HIST1H2BM,LDLR,PLA2G4C |
| BEAS-2B | NEU | 10 | HIST1H4D,LDLR,MT1F |

### SUPPLEMENTARY REFERENCES

1. Gallud, A. *et al.* Multiparametric profiling of engineered nanomaterials: unmasking the surface coating effect. *Adv. Sci.* 2002221 (2020). doi:10.1002/advs.202002221

2. Gallud, A. *et al.* Cationic gold nanoparticles elicit mitochondrial dysfunction: a multi-omics study. *Sci. Rep.* **9,** 4366 (2019).

3. Catalán, J. *et al.* In vitro and in vivo genotoxic effects of straight versus tangled multi-walled carbon nanotubes. *Nanotoxicology* **10,** 794–806 (2016).

4. Costa, P. M. *et al.* Transcriptional profiling reveals gene expression changes associated with inflammation and cell proliferation following short-term inhalation exposure to copper oxide nanoparticles. *J Appl Toxicol* **38,** 385–397 (2018).

5. Ilves, M. *et al.* Surface PEGylation suppresses pulmonary effects of CuO in allergen-induced lung inflammation. *Part Fibre Toxicol* **16,** 28 (2019).

6. Scala, G. *et al.* Multi-omics analysis of ten carbon nanomaterials effects highlights cell type specific patterns of molecular regulation and adaptation. *NanoImpact* **11,** 99–108 (2018).

7. Lund, R. J. *et al.* RNA polymerase III subunit POLR3G regulates specific subsets of polya+ and smallrna transcriptomes and splicing in human pluripotent stem cells. *Stem Cell Rep.* **8,** 1442–1454 (2017).

8. Tarasova, N. K. *et al.* Cytotoxic and Proinflammatory Effects of Metal-Based Nanoparticles on THP-1 Monocytes Characterized by Combined Proteomics Approaches. *J. Proteome Res.* **16,** 689–697 (2017).

9. Monopoli, M. P. *et al.* Physical-chemical aspects of protein corona: relevance to in vitro and in vivo biological impacts of nanoparticles. *J. Am. Chem. Soc.* **133,** 2525–2534 (2011).

10. Book, F. *et al.* Ecotoxicity screening of seven different types of commercial silica nanoparticles using cellular and organismic assays: Importance of surface and size. *NanoImpact* **13,** 100–111 (2019).

11. Shi, J. *et al.* Microsomal glutathione transferase 1 protects against toxicity induced by silica nanoparticles but not by zinc oxide nanoparticles. *ACS Nano* **6,** 1925–1938 (2012).

12. Bhattacharya, K., Kiliç, G., Costa, P. M. & Fadeel, B. Cytotoxicity screening and cytokine profiling of nineteen nanomaterials enables hazard ranking and grouping based on inflammogenic potential. *Nanotoxicology* **11,** 809–826 (2017).

13. Leek, J. T., Johnson, W. E., Parker, H. S., Jaffe, A. E. & Storey, J. D. The sva package for removing batch effects and other unwanted variation in high-throughput experiments. *Bioinformatics* **28,** 882–883 (2012).

14. Friedländer, M. R., Mackowiak, S. D., Li, N., Chen, W. & Rajewsky, N. miRDeep2 accurately identifies known and hundreds of novel microRNA genes in seven animal clades. *Nucleic Acids Res.* **40,** 37–52 (2012).

15. Robinson, M. D., McCarthy, D. J. & Smyth, G. K. edgeR: a Bioconductor package for differential expression analysis of digital gene expression data. *Bioinformatics* **26,** 139–140 (2010).

16. Cox, J. & Mann, M. MaxQuant enables high peptide identification rates, individualized p.p.b.-range mass accuracies and proteome-wide protein quantification. *Nat. Biotechnol.* **26,** 1367–1372 (2008).

17. Guo, N. L. *et al.* Multiwalled carbon nanotube-induced gene signatures in the mouse lung: potential predictive value for human lung cancer risk and prognosis. *J Toxicol Environ Health Part A* **75,** 1129–1153 (2012).

18. Bourdon, J. A. *et al.* Hepatic and pulmonary toxicogenomic profiles in mice intratracheally instilled with carbon black nanoparticles reveal pulmonary inflammation, acute phase response, and alterations in lipid homeostasis. *Toxicol. Sci.* **127,** 474–484 (2012).

19. Husain, M. *et al.* Pulmonary instillation of low doses of titanium dioxide nanoparticles in mice leads to particle retention and gene expression changes in the absence of inflammation. *Toxicol. Appl. Pharmacol.* **269,** 250–262 (2013).

20. Tilton, S. C. *et al.* Three human cell types respond to multi-walled carbon nanotubes and titanium dioxide nanobelts with cell-specific transcriptomic and proteomic expression patterns. *Nanotoxicology* **8,** 533–548 (2014).

21. Søs Poulsen, S. *et al.* Transcriptomic analysis reveals novel mechanistic insight into murine biological responses to multi-walled carbon nanotubes in lungs and cultured lung epithelial cells. *PLoS One* **8,** e80452 (2013).

22. Ronzani, C., Safar, R., Le Faou, A., Rihn, B. H. & Joubert, O. Comment on: S-nitrosoglutathione (GSNO) is cytotoxic to intracellular amastigotes and promotes healing of topically treated Leishmania major or Leishmania braziliensis skin lesions. *J. Antimicrob. Chemother.* **69,** 2300–2302 (2014).

23. Foldbjerg, R. *et al.* The toxic effects of single-walled carbon nanotubes are linked to the phagocytic ability of cells. *Toxicol. Res.* **3,** 228 (2014).

24. Poulsen, S. S. *et al.* MWCNTs of different physicochemical properties cause similar inflammatory responses, but differences in transcriptional and histological markers of fibrosis in mouse lungs. *Toxicol. Appl. Pharmacol.* **284,** 16–32 (2015).

25. Nymark, P. *et al.* Extensive temporal transcriptome and microRNA analyses identify molecular mechanisms underlying mitochondrial dysfunction induced by multi-walled carbon nanotubes in human lung cells. *Nanotoxicology* **9,** 624–635 (2015).

26. Rahman, L., Wu, D., Johnston, M., William, A. & Halappanavar, S. Toxicogenomics analysis of mouse lung responses following exposure to titanium dioxide nanomaterials reveal their disease potential at high doses. *Mutagenesis* **32,** 59–76 (2017).
